# FLCMC: Federated Learning Approach for Chinese Medicinal Text Classification

**DOI:** 10.3390/e26100871

**Published:** 2024-10-17

**Authors:** Guang Hu, Xin Fang

**Affiliations:** 1 School of Statistics and Information, Shanghai University of International Business and Economics, Shanghai 201620, China; 2 School of Computer Science, Fudan University, Shanghai 200438, China; 3 Shanghai Key Laboratory of Data Science, Shanghai 200438, China

**Keywords:** federated learning, Chinese medical text, text classification, self-attention mechanism, Adam optimizer

## Abstract

Addressing the privacy protection and data sharing issues in Chinese medical texts, this paper introduces a federated learning approach named FLCMC for Chinese medical text classification. The paper first discusses the data heterogeneity issue in federated language modeling. Then, it proposes two perturbed federated learning algorithms, FedPA and FedPAP, based on the self-attention mechanism. In these algorithms, the self-attention mechanism is incorporated within the model aggregation module, while a perturbation term, which measures the differences between the client and the server, is added to the local update module along with a customized PAdam optimizer. Secondly, to enable a fair comparison of algorithms’ performance, existing federated algorithms are improved by integrating a customized Adam optimizer. Through experiments, this paper first conducts experimental analyses on hyperparameters, data heterogeneity, and validity on synthetic datasets, which proves that the proposed federated learning algorithm has significant advantages in classification performance and convergence stability when dealing with heterogeneous data. Then, the algorithm is applied to Chinese medical text datasets to verify its effectiveness on real datasets. The comparative analysis of algorithm performance and communication efficiency shows that the algorithm exhibits strong generalization ability on deep learning models for Chinese medical texts. As for the synthetic dataset, upon comparing with comparison algorithms FedAvg, FedProx, FedAtt, and their improved versions, the experimental results show that for data with general heterogeneity, both FedPA and FedPAP show significantly more accurate and stable convergence behavior. On the real Chinese medical dataset of doctor–patient conversations, IMCS-V2, with logistic regression and long short-term memory network as training models, the experiment results show that in comparison to the above three comparison algorithms and their improved versions, FedPA and FedPAP both possess the best accuracy performance and display significantly more stable and accurate convergence behavior, proving that the method in this paper has better classification effects for Chinese medical texts.

## 1. Introduction

In the age of medical intelligence, the rapid development of health and medical big data has profoundly influenced the advancement of the medical industry. Medical text data, such as doctors’ notes, patient reports, and the medical literature, have become indispensable data assets within the healthcare sector. The continuous improvement in the quantity and quality of these data provides a solid foundation for the enhancement of medical services.

However, the particularity of medical data lies in that they encompass not only the health status and medical treatment processes of the subjects but also a wealth of sensitive individual information. If handled carelessly, this could lead to a series of societal issues, including data privacy breaches, misuse of personal information, and damage to the reputation of healthcare institutions. According to IBM’s statistics, healthcare organizations have topped the list for data breach costs for nine consecutive years, with an average loss of 6.5 million U.S. dollars [1]. Despite the enactment of regulations such as the European Union’s General Data Protection Regulation (GDPR) [2] and the California Consumer Privacy Act (CCPA) [3], reliance solely on legal constraints cannot fully prevent data leaks, especially in the era of artificial intelligence, in which the protection of personal information is particularly challenging. Therefore, it is particularly important to explore medical text mining methods that can achieve intelligent and efficient processing while protecting privacy.

Medical text classification is a key step in the processing of medical text data. It involves categorizing medical text data according to specific standards to enable automated processing of information such as patient diagnoses, treatments, and tests. This includes symptom diagnosis, drug treatment, and medical literature retrieval, which can help the medical industry better understand and utilize existing medical data, alleviate the shortage of medical resources, and improve the quality and efficiency of medical services. It can be widely applied in fields such as medical diagnosis to build intelligent medical inquiry systems or assist doctors in providing accurate and rational treatment plans. 

Early medical text classification methods primarily focused on feature extraction from medical texts, such as the bag-of-words model, the TF-IDF, the N-grams, and other common methods. However, the Chinese language typically features synonyms, antonyms, and polysemy, and single-text information is insufficient to capture its complex structure and semantic richness. 

As research has advanced, scholars have proposed medical text classification methods based on deep learning that consider the textual context, greatly improving text classification accuracy and recall rates. Nevertheless, these methods still encounter numerous difficulties in practical application and promotion. On the one hand, many single medical institutions have accumulated a small amount of text data which are of low quality and feature homogeneous characteristics, often failing to meet the training needs for deep learning-based text classification. On the other hand, due to the emphasis on protecting patient data privacy by healthcare institutions, medical information has not been shared, and data are scattered across various institutions as isolated islands, greatly reducing data usability. Additionally, Chinese medical texts contain relatively complex definitions of modern medical professional terms and a large number of medical abbreviations. Feature labels are often influenced by various factors such as doctors, medical institutions, and disease types, leading to inconsistency in data distribution across different institutions. 

In response to these challenges, federated learning offers a new approach. It allows models to be trained locally or in the cloud without sharing data, and training results are transmitted back to the local institution or cloud in real time. This technology enables an intelligent medical inquiry system that can learn quickly without the need for data movement. In recent years, the integration of federated learning with deep learning, which achieves strong privacy protection and high predictive accuracy, has proven effective in performing intelligent medical tasks. 

Although scholars have proposed federated learning methods suitable for the medical field, research on federated learning methods for Chinese medical text classification is not yet mature. The main challenge lies in the inherent medical information characteristics and semantic complexity of Chinese medical texts, as well as the differences among medical institutions in patient clinical practices, treatment measures, and demographics. The heterogeneity of medical text data stored in different centers leads to biases in the classification models built collaboratively by multiple centers, lacking sufficient generalizability. Against this backdrop, this paper proposes a federated learning method for Chinese medical text classification, named FLCMC, which connects data islands between different medical institutions, breaks down data barriers, protects data privacy, and enhances the efficiency of Chinese medical text data utilization, contributing to the innovation of intelligent medical services. The main contributions of this paper are as follows:

1. This paper proposes a Chinese medical text classification method based on federated learning, named FLCMC, which applies the federated learning framework to the task of Chinese medical text classification. It enables the construction of classification models by participating medical institutions without sharing original patient data, overcoming the limitations of existing multi-center collaborative modeling that requires data aggregation and centralized training, thus filling the research gap in federated Chinese medical text classification.
2. Regarding the issue of federated heterogeneity, this paper addresses the data heterogeneity problem present in existing Chinese medical texts. It proposes the FedPA algorithm, which introduces a self-attention mechanism in the model aggregation module to assign contribution weights to clients and adds a perturbation term in the local update module to measure the discrepancy between clients and the server. The algorithm’s excellent performance on various heterogeneous datasets has been proven through multiple experiments.
3. To enhance federated classification performance, this paper improves existing federated algorithms, such as FedAvg, FedProx, FedAtt, and FedPA, by embedding custom Adam and PAdam optimizers in the local update module. It presents three new algorithms—FedProP, FedAttP, and FedPAP—and proves their effectiveness and good generalization ability on Chinese medical text deep learning models.

In summary, this paper aims to seek a federated Chinese medical text classification model architecture with superior performance, filling the current void in federated Chinese medical text classification research. By utilizing federated learning and deep learning, the paper enhances the availability of medical text data and protects data privacy while driving deep models to learn rich semantic information from Chinese medical texts. This addresses the constraints faced by most researchers in medical text classification tasks, such as small datasets, low quality, and homogeneous features.

Due to the limitations of Chinese medical text datasets, this study only conducts an analysis on the IMCS-V2 dataset, ignoring generalization discussion on other datasets. In addition, this paper only focuses on text-type medical data. In the future, it can be extended to other Chinese medical text datasets and further explore medical multimodal federated learning. Other potential further work includes considering introducing differential privacy technology to enhance privacy protection and optimizing noise addition strategies, introducing interpretability to achieve interpretable federated learning, and exploring the combination of lightweight models and federated learning to ensure performance on resource-constrained clients.

The remainder of the paper is organized as follows: Section 2 provides an overview of relevant literature, Section 3 presents the methodology and details of the developed model, Section 4 presents the datasets, experiment details and evaluation indicators, Section 5 presents the experimental results and analysis, Section 6 presents the discussion, and Section 7 concludes the paper.

## 2. Related Work

### 2.1. Research on Medical Text Classification Methods

As the volume of medical data rapidly grows, the need for automatic classification and analysis of medical texts is increasingly urgent. Researchers around the world have explored text classification methods based on machine learning and deep learning, such as traditional methods based on feature engineering like Naive Bayes and Support Vector Machine (SVM), as well as methods based on deep neural networks like convolutional neural networks (CNNs) and recurrent neural networks (RNNs). However, current research still has some deficiencies in practicality and performance. Firstly, due to the uniqueness of medical texts, including the complexity of vocabulary and semantic polysemy, existing methods may encounter comprehension errors or information loss when processing medical texts. Secondly, most studies rely on annotated datasets, but the process of annotating medical texts is time-consuming, labor-intensive, and subject to subjectivity and inconsistency. Moreover, existing methods typically assume that the data follow independent and identically distributed (IID) statistics, neglecting the issue of data heterogeneity.

#### 2.1.1. Natural Language Processing Text Classification

In the field of text classification, research by scholars both domestically and internationally mainly includes traditional machine learning algorithms and deep learning algorithms. Text classification based on machine learning consists of three main steps: text representation, feature selection, and classifier construction. Common classification algorithms include Naive Bayes, Support Vector Machine (SVM), Hidden Markov Model (HMM), and Random Forest. However, machine learning-based text classification methods strongly depend on manually annotated features of domain knowledge and have poor performance on high-dimensional data and generalization, leading to a shift from machine learning to deep learning methods for text classification after the concept of deep learning was proposed by Hinton et al. [4] in 2006.

Deep learning-based text classification leverages the automatic text representation capabilities of deep learning models, greatly improving classification efficiency by replacing the complex manual feature engineering of traditional methods. An important intermediate step in text classification is text representation. In 2013, Google’s Mikolov et al. [5] proposed the Word2Vec word vector representation method, training the model on a corpus of 6 billion words, which promoted the application of deep learning in the NLP field. In 2014, Chen et al. [6] used a convolutional layer on top of Word2Vec’s word vectors to conduct research on text classification based on convolutional neural networks (CNNs). In 2015, Tai et al. [7] proposed the tree–LSTM model, extending LSTM to tree-structured network types to learn rich semantic representations. In 2016, Yang et al. [8] introduced a hierarchical attention network for text classification, applying the attention mechanism at both word and sentence levels to differentiate the focus on more and less important content, enhancing the interpretability of the algorithm. In 2017, Liu et al. [9] proposed a two-stage sentence encoding model, first using a mean pool on a word-level Bi-LSTM model to generate the first stage of sentence representation, followed by using an attention mechanism instead of mean pooling for the same sentence to achieve better text representation.

It is evident that these studies all use various neural network models to learn text representations. Recent studies indicate that models pre-trained on large corpora (PLMs) can avoid the need to train new models from scratch, offering significant improvements for text classification tasks. In 2018, Peters et al. [10] combined self-attention mechanisms and the Bi-LSTM model framework to propose the ELMo pre-trained language model, which dynamically represents the text semantic vectors of each word in the context, effectively addressing the problem of polysemy. Following the introduction of the Transformer structure, which relies entirely on the attention mechanism for training, PLMs with large amounts of unannotated data began to emerge in the NLP field and were fine-tuned for downstream tasks. Examples include the OpenAI GPT model proposed by Radford et al. in 2018 and the BERT model proposed by Devlin et al. [11], where BERT is a model based entirely on the bidirectional Transformer structure. Unlike Bi-LSTM, the BERT model uses a masked language model to predict words that have been randomly masked or replaced, while Bi-LSTM is limited to a combination of two unidirectional language models. BERT was the first representation model based on fine-tuning and is currently the most efficient method for text vector representation. Subsequent research has focused on improving the BERT model, such as the RoBERT model proposed by Liu et al. [12] in 2019, which is more robust than BERT; the ALBERT model by Lan et al. in 2020, which reduces memory consumption and increases training speed; and the DistilBERT model by Sanh et al. [13], which uses knowledge distillation during pre-training to reduce the size of BERT while retaining most of its training capabilities.

#### 2.1.2. Medical Text Classification

Medical text classification is an important branch of text classification tasks in Natural Language Processing (NLP). The classification of medical texts often falls into the multi-label text classification domain, meaning that texts, as a type of sequential data, usually have one or multiple category labels. Currently, there has been considerable research and notable results in the field of medical text classification both domestically and internationally. In 2015, Campillos et al. [14] achieved the classification of medical and health texts by matching pre-built rules with relevant words and sentences in queries. In 2016, Roberts et al. [15] used the K-nearest neighbors algorithm to classify resource types of medical questions. In 2017, Guo et al. [16] used the SVM algorithm for classifying Chinese medical texts. Although these methods have achieved good results, they require manual construction of classification features and struggle to capture deep semantic relationships between words within sentences.

As research has progressed, Nam et al. [17] embarked on modeling work based on RNN structures and sequence-to-sequence (Seq2Seq) architecture to capture the relevance between different text label sequences, addressing the multi-label text classification problem. In 2018, Yang et al. [18] proposed viewing the multi-label classification problem as a sequence-to-sequence generation process, introducing an SGM model based on the attention mechanism and Seq2Seq architecture. However, the Seq2Seq-based sequence generation concept considers the dependency relationship between label sequences, meaning the prediction of a subsequent label strongly depends on the generation of the previous label, potentially leading to an iterative effect of incorrect label prediction; thus, the effectiveness of this sequence structure remains debatable.

With the rise of deep learning, Du et al. [19] in 2019 proposed ML-Net, a deep learning framework for biomedical texts based on the ELMo pre-trained language model, integrating the Bi-LSTM network structure and attention mechanism to capture the contextual semantic associations of biomedical texts. In 2021, Chi et al. [20] proposed a Chinese health question classification model based on the Transformer structure, utilizing the BERT pre-trained language model to represent text word vectors and employing a topic model to obtain the text’s topic–word matrix, enhancing the model’s ability to represent the semantics of medical texts and its classification results.

In the field of Chinese medical text classification, in addition to the above-mentioned work [16,20], other related work in recent years is listed as follows. Li et al. [21] proposed a Chinese disease text classification model (DKCDM) based on medical knowledge, which enhances the representation of disease texts by introducing an external medical knowledge graph, alleviates problems such as data sparseness, and uses BiLSTM, CNN, and the attention mechanism to extract multi-faceted semantic features, achieving good classification results on the Chinese disease text dataset.

Zheng et al. [22] proposed a deep neural network learning framework based on ALBERT-TextCNN for multi-label medical text classification. This model uses the ALBERT pre-trained language model for dynamic word vector representation, introduces the TextCNN convolutional neural network model to construct a multi-label classifier for training, and effectively improves the multi-label classification effect of medical texts.

Xu et al. [23] proposed a Chinese medical text classification model, CMNN, based on the Transformer bidirectional encoder representation BERT, the convolutional neural network CNN, and the bidirectional long short-term memory BiLSTM neural network. Using BERT to train word vectors, combined with CNN and BiLSTM, it captures local potential features and contextual information and achieves good accuracy.

Zheng et al. [24] proposed a multi-label medical text classification algorithm (TLCM) based on transfer learning and ensemble learning, which uses the multi-layer bidirectional Transfomer structure inside the ALBERT model to train large-scale corpora to obtain dynamic word vector representations of text in the general domain. Through transfer learning and model fine-tuning techniques, the ALBERT pre-trained language model is used to enhance the text semantics in the medical field, and the text semantic enhancement model is input into the Bi-LSTM-CNN integrated learning module to further extract information features, achieving good classification results on the Chinese health question dataset.

Chen et al. [25] proposed a text classification method based on LSI-TF-IDF two-stage feature selection for the sensitivity classification of medical text data. Through two consecutive stages of feature reduction and feature extraction, the classification accuracy is improved, and the accuracy rate, recall rate, and F1 value are all improved, proving that this method has a better classification effect on the sensitivity classification of medical text data.

Li et al. [26] proposed an improved GRU deep learning framework, LS-GRU, for solving the problem of image report text classification. By adding a layer of LSTM at the front end of the GRU neural network to extract text features and introducing a self-attention mechanism at the back end to locate classification features, experiments show that this model has more accurate classification and higher robustness.

The above research work on Chinese medical text classification has been conducted from different perspectives, focusing on specific medical text classification models and methods, and has achieved certain results in improving the performance and effect of Chinese medical text classification. However, there are still some challenges, such as data privacy protection, data islands, and heterogeneity. Federated learning provides a new idea to solve these problems, allowing models to be trained locally without sharing data, protecting data privacy. Some researchers have carried out research on the classification of non-Chinese medical texts based on federated learning. For instance, Bharti et al. [27] put forward a non-Chinese cancer text classification system based on federated learning, which employed the federated learning framework to address the issues of privacy protection and data utilization in the healthcare system. This framework encompasses stages like data acquisition, data preprocessing, and federated learning, and utilizes machine learning models such as RNN, Bi-RNN, GRU, and LSTM for text classification, achieving certain outcomes. Nevertheless, the research on federated learning methods for the classification of Chinese medical texts is still in its infancy and requires further exploration. In view of this, this paper proposes a federated learning method for Chinese medical text classification, focusing on solving the problems of data privacy and sharing, and data heterogeneity of Chinese medical texts, filling the research gap in the federated classification of Chinese medical texts, helping to improve the utilization efficiency of Chinese medical text data, protect data privacy, and promote the innovation of intelligent medical services.

### 2.2. Research on Federated Learning Technologies

Federated learning focuses on building machine learning models using datasets from multiple medical institutions while protecting data privacy. With society’s growing emphasis on personal data privacy protection, federated learning in medical data mining has emerged and shown promising outcomes, presenting a significant practical prospect and medical value. However, most current research is centered on English medical standards and electronic health record data; studies on Chinese medical data are sparse. Moreover, in federated language modeling research, there is a common challenge known as the “impossible triangle”, which suggests that optimizing performance, efficiency, and privacy simultaneously is not feasible.

#### 2.2.1. Applications of Federated Learning in the Medical Field

In the medical field, existing federated learning research primarily utilizes medical coding data and machine learning methods to handle tasks such as the classification of medical data or the segmentation of images. This includes medical image detection, patient representation learning, and predicting hospital admissions and mortality rates. In 2018, Sheller et al. [28] collaborated with multiple medical institutions to build deep learning models within a federated framework, achieving MRI brain tumor detection and marking the first application of federated learning in clinical medical imaging. Subsequently, Lee et al. [29] used hash codes within a federated environment to compute data similarity distances for patient similarity learning across medical institutions, enabling the identification of similar patients from one hospital to another without sharing patient-level information. In 2019, Li et al. [30] proposed a community-based federated machine learning algorithm that considered patient characteristics such as gender, age, and quantity, first clustering similar patients into different communities and then predicting mortality rates and lengths of hospital stay for patients in these communities. In the same year, Liu et al. [31] applied federated learning to the patient representation task in clinical texts, constructing a two-stage federated representation model that first pre-trained a patient representation model from annotated texts using a neural network; the second stage used input features pre-trained in the first stage to train a representation model for comorbidities associated with obesity within a federated learning framework. In 2020, Vaid et al. [32] combined COVID-19 datasets from five medical institutions and utilized federated learning to improve mortality predictions for hospitalized COVID-19 patients.

In recent years, researchers have increasingly turned to federated studies based on medical IoT devices. For instance, in 2021, Chen et al. [33] proposed the FedHealth federated transfer learning framework for wearable medical devices, aggregating data from different organizations without compromising privacy security and achieving personalized federated learning through knowledge transfer. Currently, international research in the medical field has made significant progress with federated learning, concentrating mostly on structured medical data discussions such as patient age, gender, and number of hospital admissions. Research on unstructured data is also focused on medical imaging, while pure clinical records and other unstructured data remain valuable resources for machine modeling in the medical field. In a clinical setting, over 70% of information is stored as unstructured text data [31]. Therefore, federated learning for medical text data not only aids in better mining the rich semantic information within medical texts but also fills the gap in domestic Chinese medical text classification research that has not considered patient privacy protection. Building an intelligent medical system that requires no data movement and facilitates rapid learning is essential for China’s exploration of smart healthcare infrastructure.

#### 2.2.2. Optimizing Data Heterogeneity in Federated Learning

In 2021, Liu et al. [34] asserted that data heterogeneity is a significant challenge in federated language modeling, especially prominent in the medical and health domain. Initially, to address Android system update issues, Google’s McMahan et al. [35] first proposed the concept of federated learning in 2016, allowing users to train models in their systems, replacing the direct upload of data with model parameter uploads, and formally introduced the Federated Averaging (FedAvg) algorithm for experiments, demonstrating its robustness to unbalanced and non-independent and identically distributed (Non-IID) data. The core idea of the algorithm is to optimize the local stochastic gradient descent process for the data owner (client) individually and aggregate the operations on the central server. As federated learning became popular, data heterogeneity emerged as a major challenge beyond communication costs, privacy, and security. Researchers conducted a series of studies on the precision performance issues that might affect algorithms due to client data heterogeneity (Non-IID).

In 2018, Zhao et al. [36] conducted experiments with data of varying degrees of Non-IID (non-independent and identically distributed) and discovered that the FedAvg (Federated Averaging) algorithm could reduce accuracy by up to 51% on the CIFAR-10 image dataset. In 2019, Ji et al. [37] focused on the field of neural language modeling and proposed the FedAtt algorithm, which uses an attention mechanism to aggregate client models, proving to perform better than the FedAvg algorithm.

In 2020, Li et al. [38] introduced the FedProx algorithm, which improved upon the local optimization function in FedAvg by adding a perturbation term to control the discrepancy between the local models and the global model. This improved the overall convergence stability and, by dynamically adjusting the number of local iterations, ensured tolerance of system heterogeneity, reducing the impact of heterogeneous data on the whole.

Karimireddy et al. [39] proposed the Scaffold algorithm, which reduces the deviation in model updates between clients by using control variables, and demonstrated that this algorithm could use the similarity of client data to achieve faster convergence. Lin et al. [40] applied knowledge distillation techniques to accelerate model convergence, suggesting that combining the strengths of local models during the aggregation phase could produce a global model closer to the ideal training state.

It is evident that numerous scholars have made some research progress on the issue of heterogeneity in federated learning, yet the problem has not been fully resolved to date, especially concerning research in the medical field, which remains limited.

## 3. Methods

### 3.1. Basic Framework for Federated Learning in Medicine

Federated learning [35] is a distributed learning framework with privacy protection. This framework allows two or more participants to cooperate to build a common machine learning model. During the training process of the model, the training data of each participant will be retained locally. They will not leave the participating party, and the relevant information of the model can be exchanged and transmitted between the participating parties in an encrypted form, ensuring that no participating party can deduce the original data of other parties. 

Generally, suppose there are currently K medical institutions (data owners) participating in training together, denoted as ${\left\{F_{i}\right\}}_{i=1}^{K}$, and the datasets they each own are denoted as ${\left\{D_{i}\right\}}_{i=1}^{K}$. The traditional training method is to collect the data ${\left\{D_{i}\right\}}_{i=1}^{K}$ of all participants, store them in a central server, and use the centralized datasets to train the model $M_{SUM}$ on the server. The federated training method is a process in which a model $M_{FED}$ can be jointly trained without collecting the data ${\left\{D_{i}\right\}}_{i=1}^{K}$ of the participating parties. The general training process is summarized into the following five steps:

Step 1: The central server sends the initialized global model parameters to all local clients;

Step 2: The central server randomly selects clients that meet the training requirements from all local clients;

Step 3: After receiving the global model parameters, the extracted local client performs gradient update steps on the local datasets and uploads the model parameters to the central server;

Step 4: After the central server receives the model parameters uploaded by all local clients extracted in the global round, it uses the weighted average strategy to aggregate the model and sends it to the clients of each medical institution;

Step 5: Each medical institution updates its own model parameters using the latest global parameters received.

Depending on the actual application scenarios, the data of the participants often have different distribution characteristics. Based on different data distribution forms, federated learning can be divided into horizontal federated learning, vertical federated learning, and federated transfer learning. Specifically, horizontal federated learning is suitable for situations where the data of the participants have overlapping data characteristics but different data samples. Vertical federated learning is suitable for situations where the data of the participants have overlapping data samples but the participants have different data characteristics. Federated transfer learning is suitable for situations where the data samples and data features of the participants have little overlap.

### 3.2. Federated Learning Optimization and Aggregation

Federated learning optimization is essentially a distributed optimization problem. McMahan et al. [35] pointed out that it solves the following technical difficulties [35], which distinguishes it from typical distributed optimization problems.

Difficulty 1: Non-independent and identically distributed. Under the federated learning framework, the training data of a given local client are usually based on the use of mobile devices by specific users in the real world. Therefore, the local datasets of each client are non-independently distributed Non-IID data;

Difficulty 2: Imbalance. In local clients, some users may use mobile devices more frequently and in more diverse ways than other users, resulting in different numbers and characteristics of local datasets for each client;

Difficulty 3: Large-scale distribution. In a real environment, the number of client samples participating in federated optimization is very large, which is far greater than the average number of samples of each distributed client;

Difficulty 4: Different communication states. Some local client devices are occasionally offline, or the connection is slow, or local training is very expensive.

It is worth noting that in centralized training, computing costs are often concerned, and currently most solutions are to use GPU training models; in federated distributed training, communication costs dominate, and on the one hand, the client can only participate in training in a non-offline state, that is, they will only participate in a small number of update rounds every day. On the other hand, the client’s data are very small compared to the total datasets. Therefore, with the help of GPU, the computational cost in federated learning is almost negligible. Therefore, researchers in the field of federated learning focus on communication costs and how to reduce communication rounds. McMahan et al. [35] proposed the theory of federated learning optimization as follows:

In ordinary distributed learning, the objective function to be optimized is as follows:

(1) $$\underset{\theta \in R^{d}}{\min}\;f(\theta )\;where\;f(\theta )\overset{def}{=}\frac{1}{n}\sum_{i=1}^{n}f_{i}(\theta )$$

Here, for machine learning problems, $f_{i}(\theta )=l(x_{i},y_{i};\theta )$ represents the loss of predicting samples $\left(x_{i},y_{i}\right)$ using the model parameter θ, that is, the loss function.

In federated learning optimization, the loss function is usually expressed as follows:

(2) $$f(\theta )=\sum_{k=1}^{m}\frac{n_{k}}{n}F_{k}(\theta )\;where\;F_{k}(\theta )=\frac{1}{n_{k}}\sum_{i\in P_{k}}^{n}f_{i}(\theta )$$

Here, m represents the number of clients selected to participate in training, n represents the total sample size, $n_{k}$ represents the sample size of the *k*th client, and $P_{k}$ represents the sequence number set of sample individuals owned by the *k*th client. When each client obeys the settings of IID data, $E_{p_{k}}[F_{k}(\theta )]=f(\theta )$ can be derived. However, under the setting of Non-IID data, it cannot be considered that f(θ) equals to $E_{p_{k}}[F_{k}(\theta )]$, that is, any local model cannot be used as a global model.

Since $n_{k}$ has nothing to do with the model parameter θ, the gradient of the loss function can also be calculated by weighted average, that is

(3) $$\frac{\partial f(\theta )}{\partial \theta }=\sum_{k=1}^{K}\frac{n_{k}}{n}\frac{\partial F_{k}(\theta )}{\partial \theta }$$

The method of calculating gradients and updating the model using this method is called the FedSGD [35] algorithm. Among them, researchers such as McMahan [35] pointed out that the simple SGD gradient descent algorithm is sufficient to adapt to the training of some models. Therefore, in the federated learning framework, priority is given to embedding SGD into the local optimization of the client, and good results were achieved.

However, due to the high communication cost, uploading the gradient to the server every time the gradient is calculated will result in low training efficiency. In order to reduce the number of communication rounds, the client usually calculates the accumulated gradients for multiple rounds and then updates the accumulated model parameters. After incremental aggregation, it is uploaded to the server. This method is called the FedAvg algorithm [35], and it is also the most classic and widely used federated learning aggregation algorithm. The specific algorithm process is as follows:

Input: $\theta_{0}$ is the random initialization parameter; K is the client device; k is the index of the client device; C is the proportion of clients performing calculations for each global round; B is the local batch size from the partition $P_{k}$; E is the number of rounds of local model training; η is the learning rate.

Output: The final global model parameter $\theta_{t+1}$:

(1) FOR global rounds t=1,2,…;
(2) The server randomly selects C⋅K client local device $S_{t}$ in proportion C to participate in training;
(3) The server sends initialized the global model parameter $\theta_{t}$ to the selected client;
(4) Parallel computation per client $k\in S_{t}$:
  (4) $$F_{k}(\theta_{t}^{k})=\frac{1}{n_{k}}\sum_{i\in P_{k}}^{n}f_{i}(\theta_{t}^{k})$$
(5) FOR client k’s local rounds i=1,2,…,E;
(6) FOR batch b∈B;
(7) The local update parameters are
  (5) $$\theta_{t+1}^{k}=\arg\min[F_{k}(\theta_{t}^{k})]=\theta_{t}^{k}-\eta \nabla l(\theta_{t}^{k};b)$$
(8) Each client sends the updated the local parameter $\theta_{t+1}^{k}$ to the server;
(9) After server aggregation, global parameters are output:
  (6) $$\theta_{t+1}=\sum_{k\in S_{t}}\frac{n_{k}}{m_{t}}\theta_{t+1}^{k}$$

It can be seen that the idea of the classic federated aggregation algorithm FedAvg is very intuitive. The training process is divided into multiple rounds, and C⋅K local models are selected to learn the data in each round. The epoch number of the *k*th local models in a round is E and the size of batch is B, so the number of iterations is $En_{k}/B$. After a round, the parameters of all local models participating in learning are weighted and averaged to obtain the global model.

### 3.3. Federated Learning Algorithms for FLCMC

Considering the distinct heterogeneity of medical text data, this paper plans to discuss data heterogeneity and optimize it before constructing a federated learning-based medical text classification architecture. Looking at the improvements proposed by past researchers in federated learning algorithms, they can be divided into two main areas: (1) model aggregation and (2) local updates.

Ji et al. [37] focused on the scenario of English-language modeling for virtual mobile keyboards and used the FedAtt algorithm, which employs an attention mechanism to aggregate client models. This approach achieved better performance in neural language modeling tasks compared to the FedAvg algorithm.

Li et al. [38] addressed system heterogeneity and data heterogeneity by introducing a perturbation term during local model updates in client devices. This term constrains the updates to be closer to the global model, utilizing the FedProx algorithm. Ultimately, they demonstrated that this algorithm provides more stable and accurate convergence behaviors compared to FedAvg in datasets involving images and texts.

These insights underline the importance of considering both algorithm enhancements and the unique characteristics of the data involved in federated learning, especially in sensitive and heterogeneous fields like healthcare.

Inspired by the aforementioned algorithm, we introduce a self-attention mechanism to measure the weights of clients in model aggregation and introduce perturbation constraints in local updates. We propose the FedPA algorithm, which incorporates the self-attention mechanism in global aggregation and introduces perturbed gradient descent (PGD) in local updates, a method that can be seen as a variant of SGD.

Further considering the performance of real language modeling tasks and communication costs and aiming to enhance the performance of FLCMC when deploying deep neural network language models, we have built upon the foundation of FedPA. We draw from the concepts of the Adam optimizer, proposed by Kingma and Ba [41], and integrate the Adam optimizer’s adaptive gradient adjustment for learning rates and gradient moment estimation into the local updates of federated learning. We further propose a PAdam local update algorithm. This integration is designed to improve the accuracy and stability of model training.

**Figure 1:**
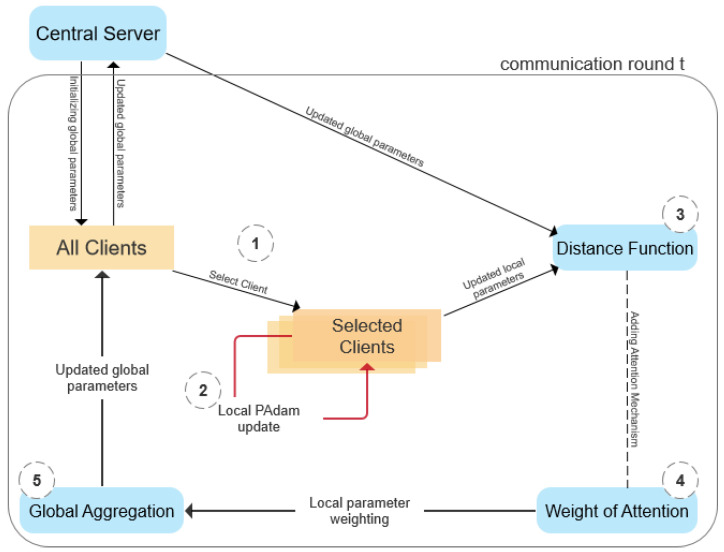
High-level view of FedPAP.

A high-level view of the proposed FedPAP algorithm is illustrated in Figure 1, which primarily includes the following two parts: (1) introducing the attention mechanism to model aggregation; (2) introducing proximal operator constraints and adaptive gradient adjustment for learning rates, along with gradient moment estimation in local updates.

#### 3.3.1. Global Aggregation

**Figure 2:**
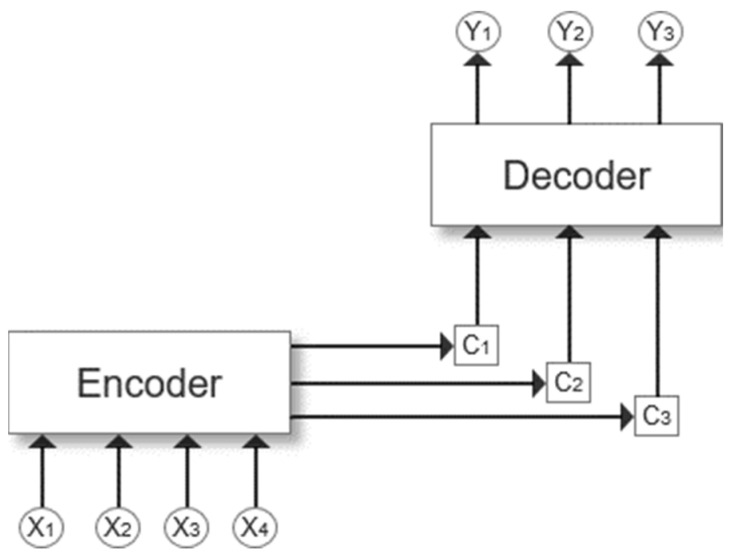
Encoder–decoder architecture diagram.

Self-attention [37,42,43,44] simulates the visual process in which humans automatically ignore non-critical information when observing things. It was originally applied to machine translation problems by Bahdanau et al. [42]. This study described attention as performing a weighted average on the encoder hidden layer to calculate decoding. The contribution of each encoder to the weighted average is determined by the similarity between the encoder’s state and the decoder’s previous hidden state. Among them, the architecture diagram of the encoder–decoder presented by Yu et al. [45] is shown in Figure 2.

The encoder–decoder architecture is also called the sequence-to-sequence problem. It is often used to deal with the problem of encoding and decoding a natural language sentence sequence to obtain a new sequence, such as multi-label text classification problems. In Figure 2, X represents the source sequence, Y represents the target sequence, and C represents the semantic encoding process, which integrates the sentence information input into the encoder, and Y is only related to C.

The role of the self-attention mechanism is to calculate the weights between each word and all other words, applying different levels of attention to different encoder word vectors and giving greater weight to the parts crucial for deciding the decoder word vectors. Less important information is given lesser weight, effectively solving the problem of long-distance dependencies in sentences. This mechanism has been widely applied to enhance RNN and CNN models. Below, the specific theory is introduced:

The self-attention mechanism treats the source text sequence processed by the encoder as a sequence of key-value pairs composed of a key vector K and a value vector V. The output vector is the weighted average of the value vector V, and the weight is determined by the similarity between the query vector Q and the key vector K. Here, the query vector Q corresponds to the query sequence predicted by the decoder as it interprets the semantics. In general, the attention function is a function that maps a query vector Q and some K-V key-value pairs into an output. The specific calculation steps are as follows:

Step 1: Calculate similarity. Calculate the similarity between the vector $Q=(q_{1},q_{2},\cdots ,q_{M})$ and the key vector $K=(k_{1},k_{2},\cdots ,k_{N})$, mainly calculating the score $e_{ii}$ of the similarity between $q_{i}$ and each $k_{i}$. The commonly used calculation function is as follows:

(7) $$e_{ii}=\left\{\begin{array}{l} q_{i}^{T}Wk_{i},common\\ q_{i}^{T}k_{i},po\mathrm{int}\\ \frac{q_{i}^{T}k_{i}}{\sqrt{d_{k}}},scalar\;dot\;product\\ h^{T}\tanh(Wq_{t}+Uk_{i}),add\;up\mathrm{sca}\\ h^{T}\tanh(W[q_{t};k_{i}]),put\;together \end{array}\right.$$

Step 2: Normalization. The softmax function is usually used to normalize the similarity score $e_{ii}$ to obtain the weight corresponding to the key, as follows:

(8) $$a_{ii}=soft\max(e_{ii})=\frac{\exp(e_{ii})}{\sum_{n-1}^{N}\exp(e_{ii})}$$

Step 3: Weighted sum. Combine the weight $a_{ii}$ corresponding to each key and its corresponding value $v_{ii}$ in the value vector $V=(v_{1},v_{2},\cdots ,v_{N})$ to perform a weighted sum to obtain the output of attention. The attention function is as follows:

(9) $$Attention(q_{i},K,V)=\sum_{i}a_{ii}v_{i}$$

The Transformer model proposed by Vaswani et al. [44] applies the attention of scaling dot products based on the efficiency of matrix multiplication compared with addition and the difficulty of gradient calculation due to large dimensions. 

**Figure 3:**
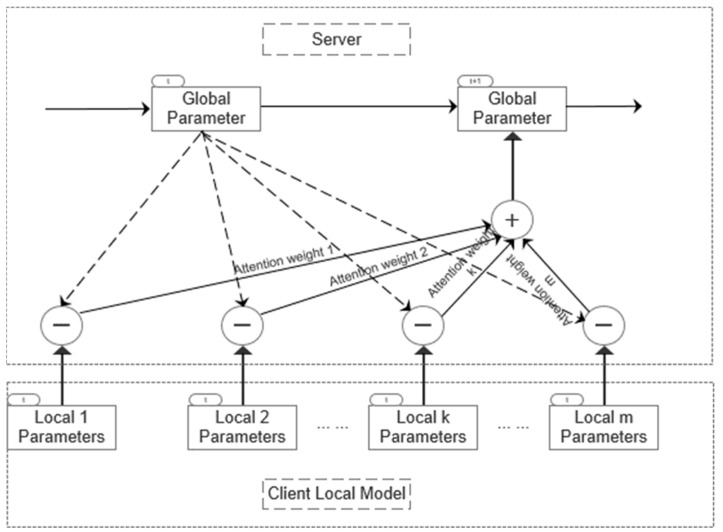
Self-attention weight federated aggregation structure diagram.

This section draws on the self-attention mechanism proposed by Ji et al. [37] to introduce the federated learning model aggregation method. The goal is to find an optimal global model close to the client model in the parameter space, that is, the input vector is the parameter vector of the client model, the output vector is the parameter vector of the server model, and the attention weight is regarded as the similarity between the server (global) model parameters and the client (local) model parameters. Here, the specific federated aggregation structure is given in Figure 3:

The figure only shows the aggregation structure separated by time steps t=1, in which the model parameters are represented by θ, the attention weight is represented by α, the number of clients is represented by m, and “+” and “−” represent parameter operations on the neural network model. Combined with the understanding of structure diagrams, the objective function of optimization is defined as follows:

(10) $$\underset{\theta_{t+1}}{\arg\min}\sum_{k=1}^{m}[\frac{1}{2}\alpha_{k}L{\left(\theta_{t},\theta_{t+1}^{k}\right)}^{2}]$$

Among them, $\theta_{t}$ is the parameter of the server model at moment t; $\theta_{t+1}^{k}$ is the model parameter for the *k*th client at moment t+1; L(,) is defined as the distance between the two sets of parameters; $\alpha_{k}$ is the attention weight used to measure the similarity between the client and the server.

Combining Formulas (7)–(9) and the method of calculating attention weight α, the corresponding specific attention weight calculation steps are as follows:

First, given the server model parameter as $\theta_{t}$, and the model parameter of the *k*th client as $\theta_{t}^{k}$, here, the L2 norm is used to calculate the similarity between parameters, that is

(11) $$s_{k}={\left\|\theta_{t}-\theta_{t}^{k}\right\|}_{2}$$

Secondly, softmax is used as a function to normalize the similarity and define the attention weight as

(12) $$\alpha_{k}=soft\max(s_{k})=\frac{e^{s_{k}}}{\sum_{k=1}^{m}e^{s_{k}}}$$

Then, combined with Equation (10), the gradient of the server model parameter $\theta_{t}$ can be calculated as

(13) $$\nabla J(\theta_{t})=\sum_{k=1}^{m}\alpha_{k}(\theta_{t}-\theta_{t+1}^{k})$$

Among them, m represents the number of clients selected in this global update round. Finally, the global parameters output by the server after attention weight aggregation can be calculated as

(14) $$\theta_{t+1}=\theta_{t}-\lambda \sum_{k=1}^{m}\alpha_{k}(\theta_{t}-\theta_{t+1}^{k})$$

Among them, λ represents the step size. It is not difficult to see that the method of calculating the global model parameters here is different from the aggregation algorithm in Equation (6), which directly weights and averages the parameters of each client based on the number of client samples. Instead, it minimizes the distance between the global parameters and the client parameters, and adds attention weights to measure the similarity between the client parameters and the server parameters.

#### 3.3.2. Local PAdam Update

In 2014, two scholars, Kingma and Ba [41], proposed the Adam optimizer, which combines the advantages of two optimization algorithms, AdaGrad and RMSProp. The update step size is calculated by comprehensively considering the first-order moment estimate of the gradient (i.e., the mean value of the gradient) and the second-order moment estimate (i.e., the uncentered variance of the gradient). The basic formula is as follows:

(15) $$g_{t}=\nabla_{\theta }J(\theta_{t-1})$$

(16) $$m_{t}=\beta_{1}m_{t-1}+(1-\beta_{1})g_{t}$$

(17) $$v_{t}=\beta_{2}v_{t-1}+(1-\beta_{2})g_{t}^{2}$$

(18) $${\hat{m}}_{t}=m_{t}/(1-\beta_{1}^{t})$$

(19) $${\hat{v}}_{t}=v_{t}/(1-\beta_{2}^{t})$$

(20) $$\theta_{t}=\theta_{t-1}-\eta \cdot {\hat{m}}_{t}/(\sqrt{{\hat{v}}_{t}}+\varepsilon )$$

First, as shown in Formula (15), the gradient of the time step *t* is calculated; as shown in Formula (16), secondly, the exponential moving average of the gradient is calculated and $m_{0}$ is initialized to 0. Similar to the momentum algorithm, this step combines the gradient momentum of historical time steps, where the coefficient $\beta_{1}$ is the exponential decay rate controlling the weight distribution (momentum and current gradient); usually, the default value is 0.9. Then, as shown in Formula (17), the exponential moving average of the gradient square is calculated, $v_{0}$ is initialized to 0, and the coefficient $\beta_{2}$ is the exponential decay rate. The default value which controls the influence of the historical gradient square is usually 0.999. Similar to the RMSProp algorithm, a weighted average of the squared gradients is performed. Because $m_{0}$ is initialized to 0, this leads to $m_{t}$ being biased towards 0, especially in the early stages of training. Therefore, as in Formula (18), it is necessary to correct the bias of the average gradient $m_{t}$. The calculation of ${\hat{v}}_{t}$ in Formula (19) is based on the same principle. Finally, as shown in Formula (20), the initialized learning rate η is multiplied by the ratio of the gradient mean to the square root of the gradient variance to obtain the updated parameters. Similar to the RMSProp algorithm, generally set $\varepsilon =10^{-8}$ and the learning rate η=0.001. $\varepsilon =10^{\wedge }(-8)$.

Combined with previous research on federated optimization algorithms, federated local optimization is usually based on the SGD algorithm. However, problems such as low training accuracy, slow convergence of the loss value, and large fluctuations in deep learning language modeling often occur [46]. Therefore, this paper is inspired by Li et al. [38] and proposes a PAdam local update algorithm based on the idea of embedding disturbance terms in SGD local update and the advantages of the Adam optimizer. The details are as follows: 

The general local client SGD gradient update is shown in Equation (5), that is, the local objective function to be updated is $F_{k}(\theta_{t}^{k})$.

This article considers adding disturbance terms locally and embedding the idea of Adam optimizer, that is:

(21) $$H(\theta_{t}^{k},\theta_{t})=F_{k}(\theta_{t}^{k})+\mu /2{\left\|\theta_{t}^{k}-\theta_{t}\right\|}^{2}$$

(22) $$g_{t+1}=\nabla H(\theta_{t}^{k},\theta_{t})=F_{k}(\theta_{t}^{k})+\mu (\theta_{t}^{k}-\theta_{t})$$

(23) $$m_{t+1}=\beta_{1}m_{t}+(1-\beta_{1})g_{t+1}$$

(24) $$v_{t+1}=\beta_{2}v_{t}+(1-\beta_{2})g_{t+1}^{2}$$

(25) $${\hat{m}}_{t+1}=m_{t+1}/(1-\beta_{1}^{t+1})$$

(26) $${\hat{v}}_{t+1}=v_{t+1}/(1-\beta_{2}^{t+1})$$

(27) $$\theta_{t+1}^{k}=\theta_{t}^{k}-\eta \cdot {\hat{m}}_{t+!}/(\sqrt{{\hat{v}}_{t+1}}+\varepsilon )$$

In this context, $\theta_{t+1}^{k}$ represents the updated model parameters of the client k at time t+1, $\theta_{t}^{k}$ is the model parameter of the client k at time t, $\theta_{t}$ is the global model parameters at time t, $H(\theta_{t}^{k},\theta_{t})$ is the objective function that includes the added perturbation term, $\mu /2{\left\|\theta_{t}^{k}-\theta_{t}\right\|}^{2}$ is the added perturbation term, and μ is a hyperparameter that constrains the difference between the local model and the global model. The remaining parameters and the corresponding default values are no different from the general settings of the Adam optimizer. 

The FedPAP algorithm proposed in this article combines a model aggregation part based on the attention mechanism with a local update part, which adds disturbance terms and incorporates Adam optimization ideas. That is, the client local update parameters obtained from Equation (27) are substituted into Formula (14), and the global model parameter is finally output, completing the construction of the entire federated optimization algorithm.

### 3.4. Datasets and Preprocessing

#### 3.4.1. Definition and Characteristics of Chinese Medical Text Datasets

Chinese medical text datasets are one category of medical and health big data, encompassing a variety of text information written in Chinese within the medical field. Medical and health big data refer to the large amounts of data generated in the medical field, with both narrow and broad interpretations. Narrowly, the term refers to big data produced by medical institutions, mainly originating from routine clinical diagnosis and treatment, research, and management processes in hospitals, including various outpatient and emergency records, hospitalization records, imaging records, diagnostic records, and medical insurance data [47]. Broadly, medical and health big data cover internet big data, regional health service platform data, disease monitoring big data, self-quantification big data, and bioinformatics data [48]. In this paper, Chinese medical text datasets are defined as symptom diagnosis records data consisting of doctor–patient dialogues, existing in an unstructured form within fields such as diagnostic results and patient complaints in doctor–patient dialogues, and these fields contain a large number of professional medical terms.

With the popularization of medical informatization and electronic medical records in our country, medical data are structured and stored in Chinese Electronic Medical Records (CEMRs), improving the readability and usability of medical information. However, in clinical practice, a large amount of electronic medical documentation is stored in unstructured and semi-structured forms, and these texts may play an important role in predicting patient risk, discovering potential disease patterns, and optimizing clinical decision-making. Domestic research on the structuring and semantic analysis of medical text data still faces challenges, mainly because, firstly, domestic research on medical informatization started late, and for a long time, there was a lack of medical text corpora. Secondly, in addition to the characteristics of traditional big data, such as volume, variety, and velocity, Chinese medical texts also possess linguistic complexity, preciseness, medical privacy security, heterogeneity, and closedness. From the perspective of textual language features, they can be summarized as follows [49]:

Word-level named entity recognition: When performing word-based named entity recognition in Chinese medical texts, the influence of segmentation may lead to changes in word properties or loss of entity information. For example, “diabetic retinopathy” might be incorrectly recognized as two entities, “diabetes” and “retinopathy”, leading to errors in named entity recognition.

Character-level named entity recognition: Although this avoids the impact of word segmentation, it does not consider word boundary information, which could potentially improve entity recognition performance. For instance, negative symptoms in medical symptom diagnosis, i.e., symptoms that do not exist or are not manifested, are significant for assessing the urgency of the condition, such as “throat pain, no itchiness in the throat” where “no itchiness in the throat” is important.

Specialty of professional terms and phrases: Chinese medical texts use a large number of professional terms and phrases with industry-specific meanings and standards; for example, in traditional Chinese medicine diagnosis, “red” corresponds to “red tongue coating” but also to “red tongue substance”. These terms and phrases may be difficult for non-medical professionals to understand.

Use of abbreviations and acronyms: In Chinese medical texts, abbreviations and acronyms are often used to replace complex Chinese names or terms when writing electronic medical records, using English acronyms instead of complex Chinese names, such as CTA (Computed Tomography Angiography), “MRI” (Magnetic Resonance Imaging), etc. However, in different contexts, these abbreviations may have multiple meanings; for instance, “CTA” might also refer to coronary angiography.

Existence of aliases and synonyms: There are many aliases and synonyms in Chinese medical texts, and so far, there is no unified medical terminology dictionary in China as a standard for writing electronic medical records. Different regions, hospitals, or doctors may use different names for the same concept, such as “Ampicillin” and “Ambicillin” or “Goji berry” and “Gouqi”.

#### 3.4.2. Chinese Medical Text Datasets

Standard datasets and richly annotated corpora are key to advancing intelligent medical development. In recent years, with the development of pre-trained language models (PTLMs) and large language models (LLMs), the usability of unannotated Chinese medical text data has greatly improved, achieving excellent training results on many downstream tasks. This section introduces commonly used public Chinese medical text datasets from recent research, summarized in the following Table 1.

By exploring the existing medical text datasets, it can be found that there are often two problems in categorizing Chinese medical text datasets in practical applications: 

(1) Lack of normalization: Influenced by the colloquial expression of the text, the same disease state may be expressed in multiple ways. For example, both “fever” and “heat” could indicate the symptom of a fever.

(2) Unknown symptom–patient relationship: It is unclear whether the patient actually has all the symptoms mentioned, as they do not necessarily possess every symptom listed in the record.

In summary, challenges such as non-unified writing standards, the small scale of annotated corpora, and the difficulty in sharing corpora still confront Chinese medical text datasets. Therefore, subsequent researchers should promote corpus sharing and improve the interpretability of unsupervised and semi-supervised learning methods while protecting data privacy and security. These efforts are essential to further develop the field of Chinese medical NLP research.

#### 3.4.3. Intelligent Conversation Medical Dataset IMCS-V2 

This paper selects the intelligent conversation medical dataset IMCS-V2 [57] for multiple NLP tasks as experimental data. This dataset collects real online doctor–patient conversations and carries out multi-level manual annotation, including named entity recognition, conversation intent, symptom labels, medical reports, etc. Compared with the original IMCS21 (V1 version) dataset, the IMCS-V2 dataset has expanded the sample size, including 4116 groups of doctor–patient dialogue case samples, covering 10 pediatric diseases. The specific data statistics are as shown in Table 2 below.

The specific content of the data consists of doctor–patient dialogue. Each sample ID corresponds to a dialogue. Each dialogue contains multiple sentences, and a random example is displayed in Table 3 below.

It can be seen from the data form that the text data corresponding to the I MCS-V2 dataset mainly include a patient self-report, dialogue, diagnosis, and treatment report. Among them, the dialogue field contains multiple sentences, and the sentence sentences corresponding to each dialogue sample example_id are different; each dialogue has different dialogue modes due to the different speaking characteristics of the patient and the doctor. At the same time, each sentence corresponds to a binary classification label, symptom_type, which indicates whether there is a symptom or not. In summary, this data form provides an ideal heterogeneous federated learning scenario.

**Figure 4:**
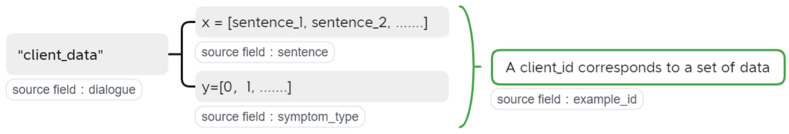
Preprocessing text data fields.

Therefore, this article targets the Chinese medical text classification task, extracts the relevant fields in the IMCS-V2 dataset that are suitable for the symptom classification task, including example_id, dialogue sentence, and symptom type, and preprocesses them into the data shown in Figure 4 below, so as to transform them into an input format suitable for federated learning.

Sentences such as sentence _1 and sentence e_2 correspond to Chinese medical segmentation texts processed by jieba. In order to ensure the accuracy of medical text segmentation, this article adds medical symptom terms compiled by the Chinese Medical Information Processing Challenge List CBLUE platform to the word segmentation database in advance. 

At the same time, in order to convert Chinese medical text into sentence vectors suitable for language model input, that is, text representation, this article plans to download the Chinese-Word2vec-Medicine pre-trained word vector from Github. The medical word vector is represented by the Word2vec method. Overall, the corpus includes medical literature, doctor–patient conversations, Wikipedia, Baidu Knows, and other medical-related corpora. The overall corpus totals 1.6 G, with a total of 7,052,948 sentences, and professional medical vocabulary is used for word segmentation.

#### 3.4.4. Synthetic Datasets

Research on federated learning heterogeneity can be divided into two aspects. One is system heterogeneity, that is, there are differences or diversity between different client devices participating in federated learning. Such differences may originate from differences in device computing capabilities, differences in network environments, and differences in device software and hardware configurations; on the other hand, there is statistical heterogeneity, which is also the heterogeneity of data distribution, that is, the distribution of data depends on the geographical location of the client, user characteristics, and data distribution differences caused by different storage rules. In this paper, the heterogeneity of data distribution is mainly studied. In order to simulate different degrees of heterogeneous data distribution forms, this paper considers using synthenic datasets to conduct algorithm evaluation experiments. synthenic datasets are mainly generated by the method proposed by Shamir et al. [59] and are widely used in federated learning experiments. The dataset consists of two parameters α and β to control the statistical heterogeneity of the generated data. Parameter α is used to control the skewness of the label distribution, that is, the larger the value of α, the greater the skewness of each client’s label distribution. Parameter β is used to control the skewness of the feature distribution, that is, the larger the value of β, the greater the skewness of the feature distribution of each client.

This article uses different α and β values to synthesize three sample datasets with 30 clients, 60 features, and 10 classes, respectively, represented as synthenic(0,0), synthenic(0.5,0.5) and synthenic(1,1), and additionally considers synthesizing dataset synthenic IID, which obeys independent and identical distribution for comparison analyze. Among them, the synthenic IID dataset is generated based on the same distribution of each client data feature and class. The final data distribution obtained is shown in Table 4 below.

**Figure 5:**
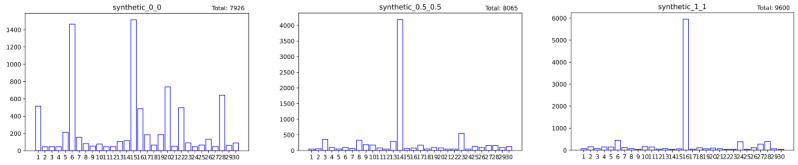
Distribution of sample size across different clients.

**Figure 6:**
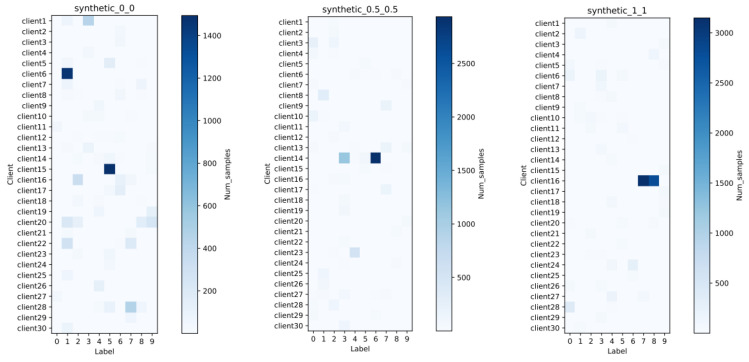
Distribution of the number of categories.

At the same time, the sample size and class distribution of each client’s training data in the three types of heterogeneous datasets are visualized in Figure 5 and Figure 6.

As can be seen from Figure 5, as the value of α and β increases, the imbalance in sample size also increases. For example, in the dataset synthenic(0.5,0.5), the 14th client with the largest sample size has 4185 samples, while the minimum sample size is only 45. As can be seen from Figure 6, although the parameter value set in the dataset synthenic(0,0) is 0, that is, there is no manual control of the skewness of data categories and features, the class imbalance in each client is also extremely large. We randomly selected Client 15 for viewing and found that there are 1494 samples in category 5, while the number of samples in category 9 is only 21. 

## 4. Evaluation

### 4.1. Comparison Algorithms

The core experimental part in this section uses three federated learning algorithms for comparison:

FedAvg [35]: This method implements model aggregation of federated learning through the averaging of model parameters. After each client is trained locally, the model will randomly select some clients and upload the updated model parameters to the server. The server then aggregates the parameters according to the sample size weight and issues them to each client.

FedProx [38]: This method is an improved federated learning algorithm that balances the differences between the local model and the global model by adding perturbation terms. It introduces a disturbance term based on the FedAvgalgorithm, making the locally updated model parameters closer to the global model, effectively improving the generalization performance and convergence speed of the model.

FedAtt [37]: This method is a federated learning algorithm with good performance in federated language modeling scenarios. It introduces a distance function to measure the difference between the client and the server in the global update module and introduces an attention weight to measure the client’s contribution during model aggregation, measuring the importance of the client and accelerating the learning process.

FedPA: The federated learning algorithms proposed in this article.

It is worth noting that the local update modules of these four algorithms are implemented by simple SGD. The local gradient update method used by the FedProx algorithm and the FedPA algorithm is essentially perturbed gradient descent (PGD) and can be regarded as a version of SGD. variant. This type of optimization method has been shown to perform poorly in the training of deep model language modeling [46]. Therefore, this paper considers embedding the Adam optimizer in the local update module of the four algorithms.

Among them, the FedAvg and FedAtt algorithms can directly call the Adam optimizer (Tf-Adam) defined in the TensorFlow library when updating the local model, but the FedProx algorithm and the FedPA algorithm proposed in this article have improved the local update module, that is, it is necessary to customize the Adam optimizer for embedding on the basis of improvement. Therefore, this article uses the TensorFlow library to customize the local update algorithm of the federated learning client, which mainly includes the custom Adam optimizer (Self-Adam) and the custom added perturbation term-constrained Adam optimizer (PAdam).

At the same time, in order to verify the reliability and effectiveness of the custom Adam optimizer, this section embeds the Tf-Adam and Self-Adam optimizers in the classic FedAvg algorithm for comparative experiments. It is intended to verify the effectiveness of Self-Adam local optimization in the experimental results, so that the comparative experiment analysis of embedding the custom optimizer into other federated algorithms is more scientific and reasonable. All federated algorithms participating in the experiment are listed below (Table 5 and Table 6).

Among them, FedPA and FedPAP are a perturbed federated learning algorithm based on the attention mechanism and the perturbed Adam federated learning algorithm based on the attention mechanism proposed in this article. The difference between the two mainly lies in the type of local update optimizer.

### 4.2. Experiment Details

All experiments in this paper were implemented using Python 3.8 and the TensorFlow 2.5.0 [60] library for machine learning. The experiments were performed on a processor equipped with a 12-core/Xeon^®^ Platinum 8255C processor and an RTX 3090 (24 GB) GPU. The floating-point arithmetic power was 35.58 TFLOPS in single precision and 71 Tensor TFLOPS in half precision.

In order to better evaluate the performance of the algorithm from a federated perspective, such as by adding disturbance terms and attention mechanism aggregation, the experiments in this section will analyze the SGD algorithm and the Adam algorithm, respectively, that is, we will compare the algorithms using the same optimizer. The same learning rate is used for the SGD algorithm experiment, and the number of clients participating in training is set to 10. The relevant hyperparameter settings are as shown in Table 7 below.

The settings of the above hyperparameters are all based on existing research on federated learning experiments conducted on the dataset synthenic as reported in studies [38,46]. The settings of these hyperparameters are based on existing research on federated learning experiments conducted on the dataset as reported in studies [38,46]. The *learning_rate* = 0.01 is identified as an optimal performance value for experiments using the SGD optimizer as determined by researchers, whereas for the Adam optimizer, it is often set to 0.001. However, adjustments might be necessary depending on the specific dataset, learning tasks, and training models involved. In this paper, to ensure fairness in the comparison of federated algorithms, the hyperparameters are uniformly set to the aforementioned optimal values. 

Additionally, the setting of the constraint difference parameter *mu* primarily references the experimental performance comparison of the FedProx algorithm. Researchers in study [38] suggest that the optimal hyperparameter value of *mu* for experiments on the dataset is set at 1. For the stepsize parameter, which constrains the attention aggregation weight in the FedAtt algorithm [37], specific values were not provided. However, based on the settings provided in the federated learning GitHub code for neural language modeling shared by researchers, an initial value of 1.2 can be determined. It should be noted, however, that this value is not necessarily the optimal parameter for the dataset.

### 4.3. Evaluation Indicators

This article plans to use the accuracy Acc of the global model relative to each client’s test set data to evaluate the accuracy of the algorithm and use the cross-entropy loss value loss of model training to evaluate the convergence of the algorithm. Among them, Acc is the average value of the accuracy measurement of the model in the client test set when the server communicates the global model to the client and is obtained by the weighted average of the number of samples in the client test set.

## 5. Results

### 5.1. Discussion with Synthetic Datasets

As can be seen from the description of the synthetic dataset in 3.4.4, the dataset models the various forms of data distribution suitable for federated learning well and takes into account the extremes of data distribution, which provides credible support for the results of the performance evaluation of the federated learning algorithm. Therefore, we prioritize the evaluation experiments of the algorithm performance on the synthetic datasets before evaluating it on the real dataset.

In order to evaluate the performance of the perturbed federated learning algorithm FedPA based on the attention mechanism and the perturbed Adam algorithm FedPAP based on the attention mechanism proposed in this article, this article plans to use a polynomial logistic regression model to perform classification training on the dataset synthenic and make a comprehensive algorithm evaluation. First, we perform hyperparameter analysis for SGD algorithms, select the heterogeneous dataset synthenic(0.5,0.5) for model training, and determine the optimal hyperparameters suitable for FedPA and FedPAP. Secondly, we perform heterogeneity analysis and select different SGD algorithms. The optimal hyperparameters are trained on data with different degrees of heterogeneity, and the performance of the algorithm under optimal parameter settings is compared. Finally, a client-side local optimization analysis is performed for the Adam algorithm, mainly analyzing the Adam optimizer embedded in the federated algorithm for its effectiveness and performance.

#### 5.1.1. Hyperparameter Analysis

**Figure 7:**
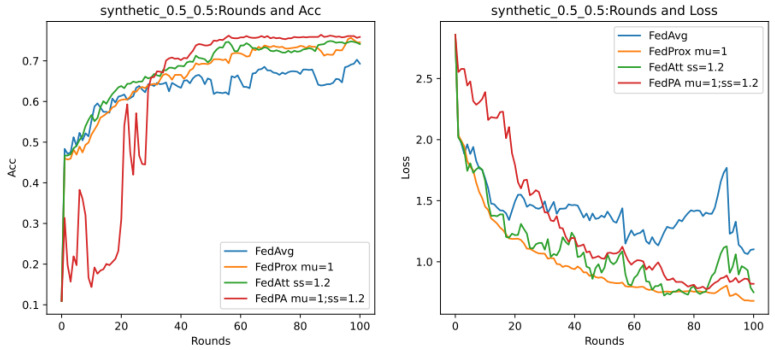
Hyperparameter default value: algorithm comparison chart.

Through previous presentations, we know that different values of the hyperparameters mu (that is, μ in Formula (21)) and stepsize (that is, λ in Formula (14)) will greatly affect the performance of the entire model, and they are also important parameters that highlight the core of the FedPA algorithm. In addition, since the core parameter that affects the different performance of the SGD algorithm and the Adam algorithm is the learning rate of the optimizer, this section aims to explore the performance differences between different algorithms, mainly focusing on the SGD algorithm, and to analyze the two hyperparameters. The default values in Table 7 are used for experiments, and the results are shown in Figure 7 below.

It can be seen that in the evaluation of Acc (accuracy), the accuracy rate of the FedPA algorithm in the later stage is higher than that of other comparison algorithms, but the Acc values of the three types of algorithms—FedAvg, FedProx, and FedAtt—basically start to rise steadily within five rounds. However, the oscillation of the FedPA algorithm is obvious in the first 30 rounds, and the convergence speed of the loss value also lags behind other algorithms. In contrast, the FedProx algorithm uses the optimal hyperparameter mu = 1 based on the dataset synthenic. Although the Acc value does not reach the optimal value, the trend in the loss value is very stable, and the performance is optimal. Therefore, this article intends to select different hyperparameter values to explore the optimal hyperparameters of the FedPA algorithm.

1. Stepsize Hyperparameter Analysis

**Figure 8:**
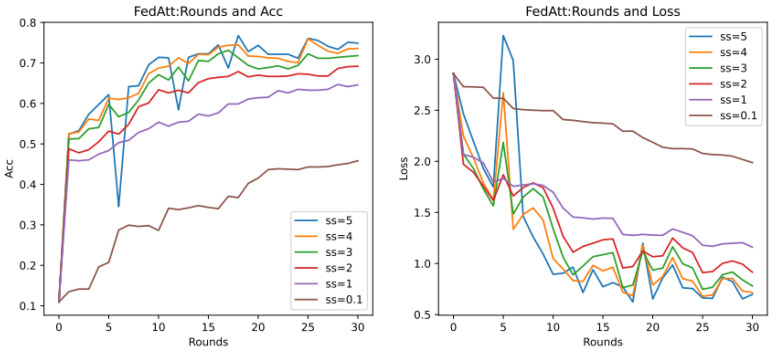
FedAtt stepsize hyperparameter: experimental result chart.

As previously described, the optimal hyperparameters of the FedAtt algorithm on the dataset synthenic cannot be determined. Therefore, in order to ensure the fairness of the comparison, priority is given to the hyperparameter analysis of the stepsize value of the FedAtt algorithm. This is also aimed at exploring the expansion of the algorithm’s performance on other datasets. This article sets the values of stepsize to 0.1, 1, 2, 3, 4, and 5, respectively, and the number of communication rounds is 30. The results are shown in Figure 8 below.

In the figure, the parameter stepsize is abbreviated as ss. It can be seen that the Acc value of FedAtt increases with the increase in stepsize between 1 and 4. When stepsize = 5, the Acc value fluctuates significantly, and it is not much improved compared to stepsize = 4, with a similar convergence trend; when stepsize = 0.1, the performance is the worst. Therefore, stepsize = 4 is selected as the optimal parameter for the dataset.

**Figure 9:**
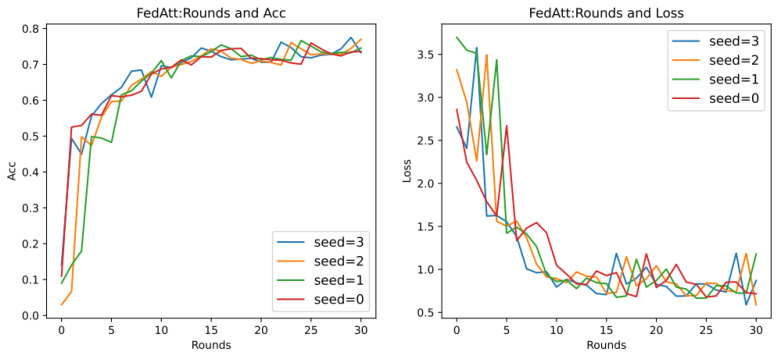
FedAtt seed hyperparameter: experimental result chart.

At the same time, in order to avoid the randomness of the experimental results and reduce the sampling error of the experiment, this section plans to set different random seeds and repeat the same experiment with stepsize = 4 mentioned above. Among them, the parameter seed controls the sample results randomly assigned to each client when synthesizing the dataset in this article. It can be understood as a dataset with the same degree of heterogeneity and different data samples. Therefore, here, the values of seed are set to 0, 1, 2, and 3, respectively, and the results are shown in Figure 9 below.

**Figure 10:**
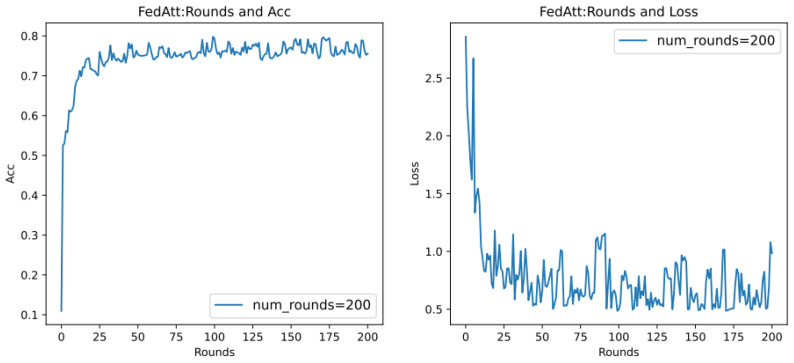
FedAtt num _rounds hyperparameter: experimental result chart.

It can be seen that, on different datasets, the effect of Acc is pretty good, and the loss value basically shows a unified trend. It only differs in the time points of the fluctuations of different datasets, indicating that the dataset has a certain impact, and the loss value will follow a certain convergence trend. Therefore, in order to observe the convergence trend in loss, the number of communication rounds will be extended to 200 rounds (Figure 10) (original: 30 rounds). 

It can be seen that when the number of communication rounds num_rounds is about 25, even if the number of communication rounds is increased, the Acc performance and loss value of the model do not significantly improve or decrease. At this time, the model can be considered to have converged.

In summary, it is determined that the optimal hyperparameter stepsize of FedAtt in the dataset is 4.

2. mu Hyperparameter Analysis

**Figure 11:**
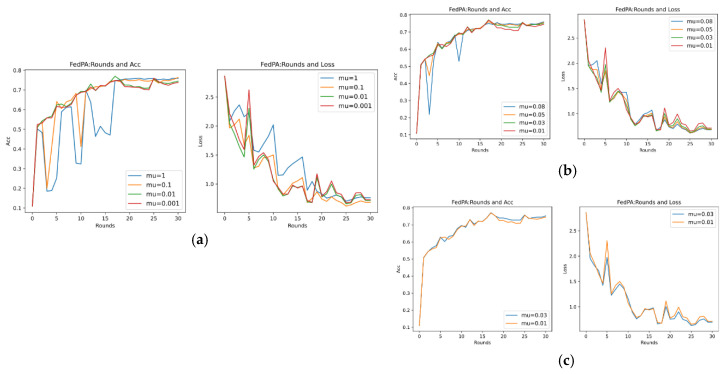
FedPA mu hyperparameter: experimental result chart. (**a**) mu set to 0.001, 0.01, 0.1 and 1; (**b**) mu set to 0.01, 0.03, 0.05 and 0.08; (**c**) mu set to 0.01and 0.03.

As seen in Table 5, the FedPA algorithm is essentially a combination of FedProx and FedAtt. In federated learning model aggregation, the FedPA algorithm uses an attention mechanism similar to the FedAtt algorithm. Therefore, here, we first assume that the optimal stepsize hyperparameter of FedPA on synthenic the dataset is 4, and analyze the impact of different values of the Mu hyperparameter. Researchers such as Li [38] used different values as references when exploring the influence of the value of *mu* and set the values to 0.001, 0.01, 0.1, and 1. The results are shown in Figure 11a below.

As seen in Figure 11a, mu = 0.01 and mu = 0.001 perform better. When the mu value is large, both the accuracy performance and loss have large oscillations. However, when the mu value is too small, the advantages of the disturbance term cannot be fully utilized. When mu = 0.1, it shows a relatively excellent effect on loss, but the Acc performance is not satisfactory, so we continue to use mu = 0.01~0.1 to make more precise values, which are 0.01, 0.03, 0.05, 0.08. It can be seen from the result in Figure 11b that mu = 0.08 is no different from mu = 0.1, and the effect of mu = 0.03 or mu = 0.01 is better. As can be seen from Figure 11c, whether looking at Acc or loss, it can be determined that mu = 0.03 has the best effect, so the optimal parameter mu = 0.03.

As can be seen in Figure 10, and a stepsize value experiment of the FedPA algorithm under the optimal parameter mu was carried out. In summary, it was determined that, for the FedPA algorithm on the dataset, the optimal stepsize = 4 and the optimal mu = 0.03.

#### 5.1.2. Heterogeneity Analysis

**Figure 12:**
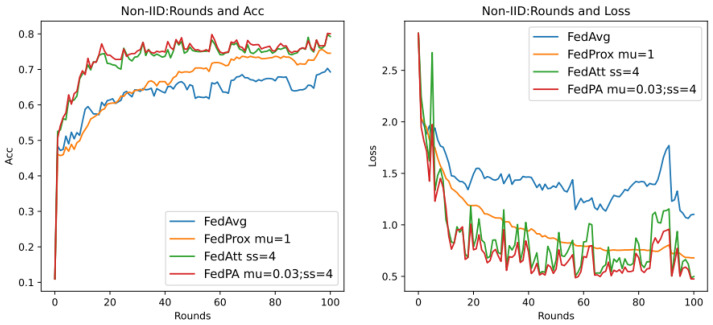
SGD algorithm comparison result chart.

This section discusses the performance of the four SGD algorithms on data with different degrees of heterogeneity, prioritizes the selection of optimal parameters for the four algorithms for model training, and makes comparative experimental results as shown in Figure 12 below.

Through the comparison of four algorithms, it is found that FedPA performs best in terms of both Acc and loss. In terms of a specific analysis, the performance of the FedAtt algorithm and the FedPA algorithm in Acc is similar, but the FedPA algorithm shows a better trend in terms of the stability of loss convergence. At the same time, only experiments comparing the performance of the FedAtt algorithm compared to the FedAvg algorithm have been conducted in the field of language modeling before. This section will also further expand the research on the heterogeneous performance of the FedAtt algorithm.

**Figure 13:**
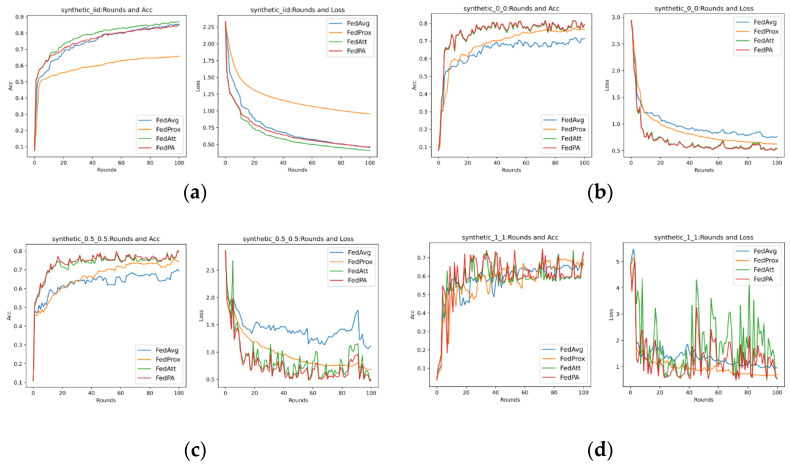
Comparison results of different heterogeneous datasets. (**a**) *synthenic IID*; (**b**) *synthenic*(0, 0); (**c**) *synthenic*(0.5, 0.5); (**d**) *synthenic*(1, 1).

Next, this section conducts comparative experiments of four algorithms on synthenic IID, synthenic(0,0), synthenic(0.5,0.5), and synthenic(1,1), four types of datasets with different degrees of heterogeneity. The results are shown in Figure 13 below.

Through the comparison of four algorithms, it was found that on the independent and identically distributed synthenicIID dataset, FedProx performed the worst, and the other three algorithms performed similarly. Unexpectedly, it was found that the performance of the FedAtt algorithm was slightly better, while the performance of the FedPA algorithm was similar. It was slightly better than that of the classic FedAvg algorithm. Observing the algorithms’ performance of the heterogeneous datasets synthenic(0,0), synthenic(0.5,0.5), and synthenic(1,1), it was found that the FedPA algorithm performed best on moderately heterogeneous datasets, and its loss convergence was more stable than that of the FedAtt algorithm. On extremely heterogeneous datasets, the oscillations of the algorithm are relatively obvious. Overall, FedPA performs best on moderately heterogeneous datasets.

#### 5.1.3. Client Local Optimization Analysis

This section focuses on exploring the effectiveness and performance of the Adam optimizer embedded into the federated learning framework and also lays the foundation for subsequent training of federated language models with guaranteed performance in Chinese medical texts. There are three types of Adam optimizers involved in the experiment: Self-Adam, Tf-Adam, and PAdam. The principles of Self-Adam and Tf-Adam are the same. The only difference is that Tf-Adam is directly provided by the TensorFlow library. Self-Adam is the optimizer customized in this article. The comparison between the two aims to verify the Self-Adam optimization. The effectiveness of the optimizer also paves the way for the perturbation PAdam optimizer based on the Self-Adam improvement.

1. Effectiveness analysis

In order to explore the effectiveness of the Adam optimizer embedded in federated learning, this article intends to conduct a comparative analysis on the Adam-type algorithm and the SGD-type algorithm on the heterogeneous dataset synthenic(0.5,0.5), that is, to explore FedAvg vs. FedAvgS vs. FedAvgT, FedProx vs. FedProxP, FedAtt vs. FedAttS, and FedPA vs. FedPAP.

**Figure 14:**
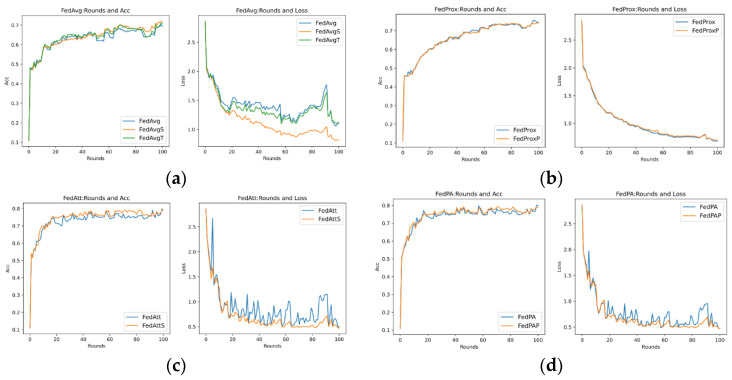
Experimental analysis of effectiveness of Adam algorithm. (**a**) FedAvg vs. FedAvgS vs. FedAvgT; (**b**) FedProx vs. FedProxP; (**c**) FedAtt vs. FedAttS; (**d**) FedPA vs. FedPAP.

It is important to note here that the learning rate of the optimizer is uniformly set to 0.001 because of the previously mentioned optimal learning rate of Adam, learning rate = 0.001. The experimental results are shown in Figure 14 below.

Among them, the indicators for comparing different algorithms in the figure are still the Acc value and loss value (the horizontal axis represents the number of communication rounds num_rounds and the vertical axis represents the Acc value and loss value). It can be seen that the local model based on the experiments in this chapter is a relatively simple logistic regression model, and the performance improvement derived from embedding the Adam optimizer is not significant. However, when observing Figure 14a, it is found that FedAvgS has a significantly superior performance in loss convergence, which proves the effectiveness of the Self-Adam optimizer. At the same time, by observing the FedAtt class algorithm and the FedPA class algorithm, it can be found that the Self-Adam optimizer and the embedding of the PAdam show good loss convergence stability while ensuring Acc performance. This is also consistent with the advantage of the parameter updates in the Adam optimizer proposed by Kingma and Ba [41], which are not affected by the scaling transformation of the gradient and can significantly reduce oscillations.

In summary, the effectiveness of this article’s customized Self-Adam optimizer and PAdam optimizer is proven.

2. Performance analysis

**Figure 15:**
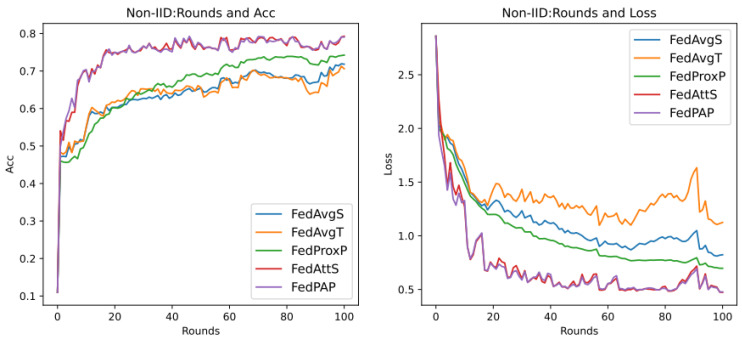
Adam algorithm performance: test analysis.

This section mainly compares different Adam-type algorithms to explore the differences in the performance of various Adam-type algorithms. The results are shown in Figure 15 below.

It is not difficult to see that the federated learning algorithms FedPAP and FedAttS that embed the attention mechanism into model aggregation have shown outstanding advantages. The accuracy value, Acc, is significantly improved compared to other algorithms. For better comparison, the maximum Acc value and minimum loss value of various algorithms are listed in Table 8 below.

It can be seen that the best loss value can be reduced by 0.63 compared with the worst loss value. At the same time, given that the mu parameter setting of FedPAP is small, the disturbance term has little effect, and the classification model used is relatively simple, the Acc performance and loss convergence are basically consistent with the performance of FedAttS. Subsequent studies will be carried out on real datasets and further observe the performance difference between the two in deep learning models.

### 5.2. Discussion of Chinese Medical Text Datasets

This section proposes to construct a joint learning framework for real-world Chinese medical texts, aiming to solve the existing problems of data privacy, data silos, and data heterogeneity in the medical field and to fill the research gaps in the application of joint learning for Chinese medical texts. This section further explores the generalization ability of the joint algorithm on real-world datasets (i.e., Chinese medical text dataset) and deep learning language models.

The experimental results and analysis presented in this article are divided into three parts. First, due to variations in datasets, the optimal hyperparameters for the FedPA algorithm and the FedPAP algorithm require further discussion. Secondly, to assess the performance of the SGD and Adam algorithms on the Chinese medical text dataset, a logistic regression model is employed for experimental analysis. Finally, LSTM text classification experiments are conducted using the federated learning approach, thereby verifying the federated learning Chinese medical text classification approach proposed in this article.

#### 5.2.1. Hyperparameter Analysis

The hyperparameter experimental procedure in this section is consistent with Section 5.1.1, determining the optimal hyperparameter stepsize = 9, while the experimental results regarding the optimal hyperparameter mu are shown below.

**Figure 16:**
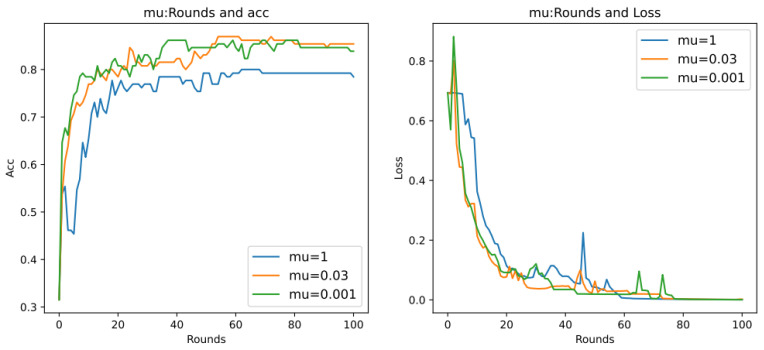
FedPAP mu hyperparameter: experimental result chart.

Based on the experimental results in Figure 16, the optimal hyperparameter mu = 0.03 for FedPAP is finally determined. Meanwhile, we further analyze the optimal hyperparameters of FedAtt and FedProx on the IMCS-V2 dataset and find that the optimal hyperparameter of FedAtt is stepize = 4, while the optimal hyperparameter of FedProx is mu = 0.03. The experimental results on finding the optimal hyperparameters of FedAtt are shown below.

**Figure 17:**
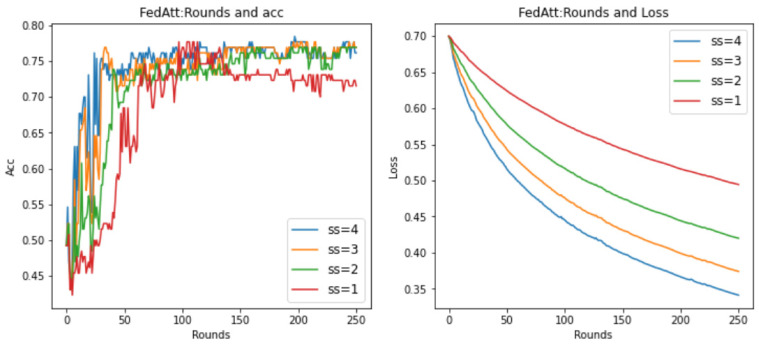
FedAtt stepsize hyperparameter: experimental result chart.

**Figure 18:**
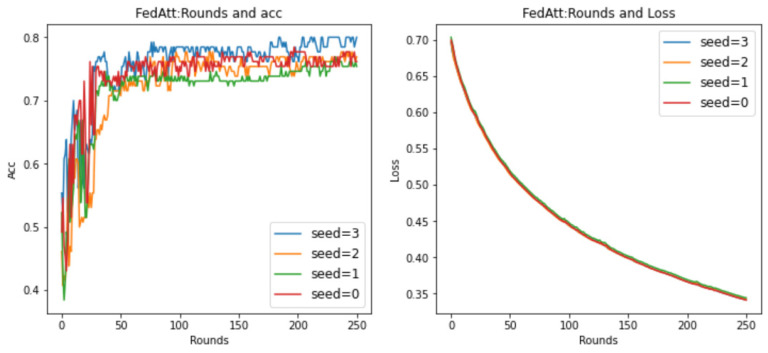
FedAtt seed hyperparameter: experimental result chart.

As can be seen from Figure 17, the experimental effect is significantly improved at stepsize = 4, and as can be seen from Figure 18, when stepsize = 4, the performance is basically the same on different randomized datasets, indicating that the trend is not a small probability event. 

#### 5.2.2. Logistic Regression Model Analysis

**Figure 19:**
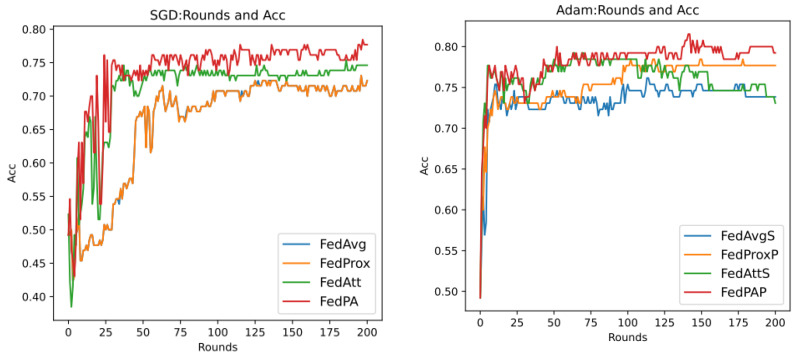
Logistic regression model classification performance results chart.

This section extends the number of training rounds to 200 and shows the classification accuracy of different federated algorithms as follows (Figure 19):

It can be seen that in the comparison of SGD algorithms, the Acc performance of the FedPA algorithm is the best, followed by the FedAtt algorithm, while the performance effects of the FedProx algorithm and the Fed A vg algorithm are basically the same; among the algorithms, in the Adam class, the FedPAP algorithm proposed in this article still has the best performance, followed by the FedProxP algorithm. The Acc value of the FedAttS algorithm has a downward trend in the later period, which shows that the performance is not stable. The maximum Acc value of the two types of algorithms is compared and expressed in Table 9.

#### 5.2.3. Deep Learning LSTM Model Analysis

In previous experiments, this paper built a logistic regression classification model for testing and verified the performance of the proposed federated algorithm on the Chinese medical text dataset. Next, this article plans to build a federated learning Chinese medical text classification framework that guarantees performance and conducts comparative analyses of SGD-like algorithms, Adam-like algorithms, and communication efficiency.

1. SGD Algorithm Analysis

**Figure 20:**
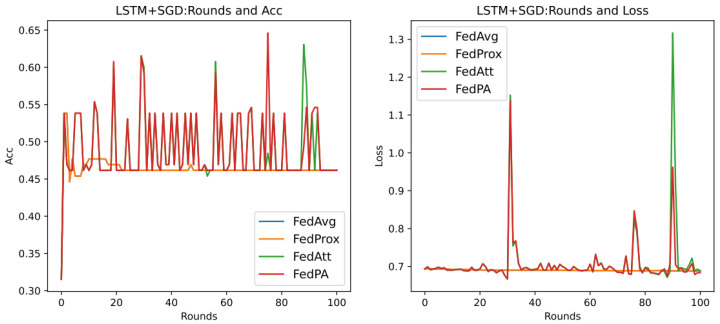
LSTM model + SGD classification: performance result chart.

In this section, learning rate = 0.01, stepsize = 9, and mu = 0.03 are set. The remaining hyperparameter settings are consistent with Table 7. The test results are shown in Figure 20 below.

As can be seen in the above figure, the performance of the FedAvg algorithm and the FedProx algorithm, as well as that of the FedAtt algorithm and the FedPA algorithm, is basically the same, indicating that in the deep learning model, there is little difference in the performance of SGD local optimization based on adding perturbation terms. At the same time, it can be observed that the Acc value fluctuates between 0.55 and 0.65, and the loss value fluctuates between 0.7 and 1.3. That is, the Acc performance is not as good as the results of the simple logistic regression model, and the loss is not presented. There is a convergence trend. This does not rule out the fact that the SGD optimizer is highly sensitive to the initial learning rate and is prone to falling into the local optimal solution, but it also shows that the applicability of SGD local optimization in federated language modeling is not high, which is also verified in the analysis of federated experimental results by Palihawadana et al. [46].

2. Adam Algorithm Analysis

**Figure 21:**
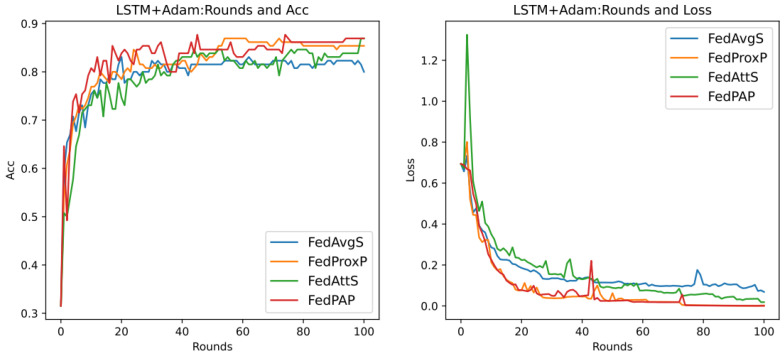
LSTM model + Adam classification: performance result chart.

In view of the problem of poor results of SGD algorithms applied in federal LSTM text classification, this section considers using the Adam type algorithm in Chapter 4 to comparatively analyze its performance in the Chinese medical text classification task. The results obtained are shown in Figure 21 below.

At the same time, the maximum Acc value and minimum loss value of various algorithms are organized as shown in Table 10 below.

The FedPAP algorithm proposed in this article is the superior algorithm in terms of Acc performance and loss convergence. At the same time, the FedProxP algorithm improved by Adam embedding shows good performance, second only to the FedPAP algorithm. This is in great contrast to the modeling results of the logistic regression model. That is, the federated learning architecture with the deep learning model and the Adam optimizer significantly improve the performance of Chinese medical text classification. At the same time, this also proves the good generalization ability of the FedPAP algorithm to deep learning language models.

#### 5.2.4. Communication Efficiency Analysis

The communication efficiency problem is another major difficulty in federated learning, in addition to the heterogeneity problem. Especially with the rapid development of large language models, many researchers in the field of federated language modeling focus on the issue of efficiency improvement, such as introducing knowledge distillation technology into federated learning [40]. However, performance and efficiency often cannot be optimized at the same time, which is the “impossible triangle” problem mentioned in this article.

The focus of this article is to improve the performance of federated Chinese medical text classification. However, in view of the high focus of federated language modeling on communication efficiency, this article intends to draw on the communication efficiency evaluation index proposed by researchers such as Caldas [61] and present the average time (in milliseconds) used for one round of communication by the Adam-type federated algorithm on the IMCS-V2 dataset as follows (Table 11).

It can be seen that the federated algorithm that adds disturbance terms to the client’s local update module overcomes the performance degradation problem caused by heterogeneity to a certain extent, but it also sacrifices communication efficiency and increases communication time. However, it can still be seen that the federated algorithms FedAttS and FedPAP, which increase the attention aggregation weight, learn the optimal global model through good generalization on each client model, reducing the number of local training rounds. This accelerates the learning process and shows superior communication efficiency than ordinary weighted aggregation federated algorithms FedAvgS and FedProxP.

## 6. Discussion

Due to the constraints of publicly available Chinese medical text datasets, this study only performs empirical analysis on the IMCS-V2 type Chinese medical text dataset, overlooking the discussion on generalization across other datasets. Future research could consider expanding to other Chinese medical text datasets to validate the algorithm’s effectiveness and robustness in broader contexts. In this study, we focused only on text data. In practice, medical data are often multimodal. They could also include medical images such as X-rays and CT scans, physiologic readings such as ECG, or patient records in a structured form. Future research could further explore multimodal federated learning approaches, thus providing a more comprehensive framework for medical diagnosis. 

Although federated learning keeps data on local nodes and avoids centralization, the information from model updates may still reveal sensitive individual data. Future research could consider incorporating differential privacy techniques to further enhance privacy protection. Differential privacy, by adding noise during model updates, effectively prevents sensitive information leakage. The trade-off between model accuracy and privacy is an important issue mentioned by Lee J et al. [29]. Noising to preserve privacy can potentially affect the federated model performance. In addition, adding differential privacy in large-scale and real-time medical systems will increase the computational load, and this side effect will reduce the overall efficiency of the system. Future studies could explore optimizing noise addition strategies to maintain high levels of privacy protection without significantly compromising model performance.

Another possible future research direction is to introduce interpretability and realize interpretable federated learning. In traditional federated learning, multiple participants jointly train models, but the decision-making process of the model is often opaque. Interpretable federated learning aims to solve this problem by introducing interpretability to make the decision-making process of the federated learning model more transparent and understandable. This is of great significance in the medical field.

While pre-training and fine-tuning techniques dominate most NLP tasks, pre-trained language models like BERT and GPT significantly enhance downstream model performance. However, in a federated learning setting, it is often impractical to deploy large models like GPT on local clients. Techniques such as knowledge distillation may offer potential solutions, but whether the simplified models can maintain performance remains an open question. Future work could further explore the integration of light-weight models with federated learning to ensure good model performance on resource-constrained clients.

## 7. Conclusions

The rapid advancement of medical informatization presents significant challenges in data privacy protection and the deployment of intelligent precision medicine. Concurrently, traditional research on medical texts is hampered by critical issues including data privacy, data silos, and heterogeneity, with the spread of data across various institutions and regions complicating their effective sharing and utilization. In response, federated learning has emerged as a promising solution. This approach involves training models on centralized servers or cloud platforms and transmitting model parameters to distributed local clients, thus ensuring data privacy while fostering the rapid development of medical treatments. This research makes several contributions, which are detailed below.

This article first provides a comprehensive review of Chinese medical text datasets, text classification methods, and cutting-edge federated learning technology in recent years, summarizing the shortcomings of existing research. Secondly, it focuses on the field of Chinese medical text privacy protection and data sharing, analyzing the more prominent problem of data heterogeneity in federated learning. Further exploration led to the proposal of the perturbed federated learning algorithm, FedPA, based on the self-attention mechanism, and the FedPAP algorithm, which also utilizes the self-attention mechanism and integrates the perturbed PAdam optimizer. In the model aggregation module, we consider combining the self-attention mechanism to assign weights to client contributions, adding perturbation terms to the local update module, and integrating the custom PAdam optimizer. This represents the first attempt to combine the attention mechanism, perturbation terms, and the Adam optimizer into a federated learning algorithm. Then, to fairly compare the performance of the proposed algorithm, the existing federated algorithm was enhanced by embedding the custom Adam optimizer. After an experimental analysis of hyperparameters, heterogeneity, effectiveness, and other factors, the federated learning algorithm proposed in this article was found to have better classification performance and convergence stability. Finally, based on the proposed algorithm, the federated learning approach for Chinese medicinal text classification was verified, and performance comparison and communication efficiency analyses of the algorithm were conducted. These analyses demonstrated that the proposed algorithm effectively improves the generalization ability of deep learning models in Chinese medical texts, further contributing to domestic research on federated Chinese medical text classification. Potential future work includes expanding Chinese medical text datasets, exploring multimodal federated learning, introducing differential privacy techniques, introducing interpretable federated learning, and exploring the combination of lightweight models and federated learning, etc.

## Figures and Tables

**Table 1:** Chinese medical text datasets.

Dataset Name	Data Volume	Data Tags	Data Type
CMeEE [50]	23,000	9 types of entities	Chinese medical text named entity recognition
CMelE [50]	22,406	53 types of relations	Chinese medical text entity relationship extraction
CMedCausal [51]	3000	3 types of relations	Medical causality relationship extraction
CHIP-CDEE [51]	2485	4 types of attributes	Clinical discovery event extraction
CHIP-CDN [50]	18,000	2500 standardized	Clinical terminology normalization
CHIP-CTC [50]	40,644	44 categories	Clinical trial screening criteria short text classification
CHIP-MEDFNPC [52]	8000	4 types of attributes	Medical dialogue clinical findings polarity determination
CHIP-STS [50]	30,000	2 categories	Ping An Healthcare and Technology Disease Q&A transfer learning
KUAKE-IR [53]	104,000	10 relevant data items	Medical paragraph retrieval
KUAKE-QLC [54]	10,880	11 categories	Medical search query intent classification
KUAKE-QTR [52]	32,552	4 categories	Medical search page title relevance
KUAKE-QQR [52]	18,196	3 categories	Medical search query relevance
MedDG [55]	22,162	160 types of entities	Chinese medical dialogue
DiaKG [56]	22,050 entities, 6890 relation triples	15 types of entities, 10 types of relations	Diabetes domain entities and relations
IMCS [57]	4116	16 types of intents, 5 types of entities, 3 types of symptoms	Intelligent dialogue diagnosis and treatment
TCMRelExtr [58]	16,150 summaries	4 types of entities, 5 types of relations	Traditional Chinese medicine entity and relationship corpus

**Table 2:** IMCS-V2 doctor–patient dialogue dataset statistics.

Statistical Indicators	Accurate Value
Total number of diseases	10
Total conversations	4116
Total number of sentences	164,731
Average number of sentences per conversation	40
Average number of characters per conversation	523
Average number of characters per conversation (including patient self-report)	580

**Table 3:** IMCS-V2 doctor–patient dialogue data format.

Main Field Name	Subfield Name	Field Explanation	Example Content
example_id	none	Conversation sample id	10001003
diagnosis	none	Patient disease category	Indigestion in children
self-report	none	Patient reports	“My baby is 16 days old and her belly is always very bloated. She still needs to feed, and she farts very hard. How should I deal with it?”
dialogue	sentence_id	Dialogue turn number	1
	speaker	doctor or patient	Doctor
	sentence	Current conversation text content	“Hello, babies who are 16 days old usually have a bigger belly after feeding. Is it good for the baby to feed? Is the breast milk enough? Is there any diarrhea?”
	dialogue_act	conversational intent	Ask about symptoms
	symptom_norm	Symptom terms that appear	Diarrhea
	symptom_type	Symptom Category	1
	local_implicit_info	Sentence symptoms and category labels	Diarrhea: 1
implicit_info	Symptom	Conversation symptoms and category labels	Diarrhea: 1; sleep disturbance: 1
explicit_info	Symptom	Self-reported symptoms and category labels	Diarrhea, fart
report	none	Diagnosis and treatment report	Chief complaint: abdominal distension History of current illness: “The 16-day-old child suffers from abdominal distension, crying easily, burping easily after feeding, and having bowel movements 6–7 times a day”. Auxiliary inspection: none Past history: none Diagnosis: indigestion in children Suggestion: “Keep warm, rub the child’s belly clockwise; take an appropriate amount of Mommy’s Love, half a pack at a time…”

**Table 4:** Dataset description.

Dataset Name	Train Dataset Size	Test Dataset Size	Number of Clients
synthenic(0,0)	7926	897	30
synthenic(0.5,0.5)	8065	912	30
synthenic(1,1)	9600	1084	30
synthenic IID	7102	805	30

**Table 5:** Description of SGD federation algorithms.

Algorithm	Model Aggregation Methods	Local Update Method
FedAvg	weighted average aggregation	SGD optimizer
FedProx	weighted average aggregation	PGD optimizer
FedAtt	attention mechanism aggregation	SGD optimizer
FedPA	attention mechanism aggregation	PGD optimizer

**Table 6:** Description of the Adam federated algorithm.

Algorithm Name	Model Aggregation Methods	Local Update Method
FedAvgS	weighted average aggregation	Self-Adam optimizer
FedAvgT	weighted average aggregation	Tf-Adam optimizer
FedProxP	weighted average aggregation	PAdam optimizer
FedAttS	attention mechanism aggregation	Self-Adam optimizer
FedPAP	attention mechanism aggregation	PAdam optimizer

**Table 7:** Hyperparameter settings.

Hyperparameters	Explain	Default Value
learning_rate	Optimizer learning rate	0.01
num_rounds	Communication rounds	1 00
clients_per_round	Number of clients per training round	1 0
num _epochs	Number of local training rounds	1 0
num_iters	Number of iterations per round	2 0
eval_every	Evaluate every few rounds	1
batch_size	Batch sample size	1 0
see	random seed	0
mu	Constraint difference parameters μ	1
stepsize(ss)	Constrained attention aggregation weight parameters λ	1.2

**Table 8:** Comparison of maximum Acc and minimum loss of Adam-type algorithms.

Algorithm Name	Maximum Acc Value	Minimum Loss Value
FedAvgS	0.72	0.81
FedAvgT	0.71	1.1
FedProxP	0.74	0.70
FedAttS	0.79	0.47
FedPAP	0.79	0.48

**Table 9:** Logistic regression model algorithm performance comparison.

Algorithm Name	SGD Class Maximum Acc Value	Adam Class Maximum Acc Value
FedAvg vs. FedAvgS	0.73	0.76
FedProx vs. FedProxP	0.73	0.78
FedAtt vs. FedAttS	0.75	0.79
FedPA vs. FedPAP	0.78	0.82

**Table 10:** Performance comparison of LSTM model + Adam algorithm.

Algorithm Name	Maximum Acc Value	Minimum Loss Value
FedAvgS	0.8308	0.0684
FedProxP	0.8692	0.0010
FedAttS	0.8692	0.0184
FedPAP	0.8769	0.0005

**Table 11:** Comparison of communication efficiency of Adam-type federated algorithms.

Algorithm Name	Average Communication Time
FedAvgS	5816.91
FedProxP	8993.86
FedAttS	5762.10
FedPAP	8627.51

## Data Availability

The original contributions presented in the study are included in the article, further inquiries can be directed to the corresponding author.
